# Least-Squares Fitting of Multidimensional Spectra to Kubo Line-Shape Models

**DOI:** 10.1021/acs.jpcb.1c08764

**Published:** 2021-11-16

**Authors:** Kevin C. Robben, Christopher M. Cheatum

**Affiliations:** Department of Chemistry, University of Iowa, Iowa City, Iowa 52242, United States

## Abstract

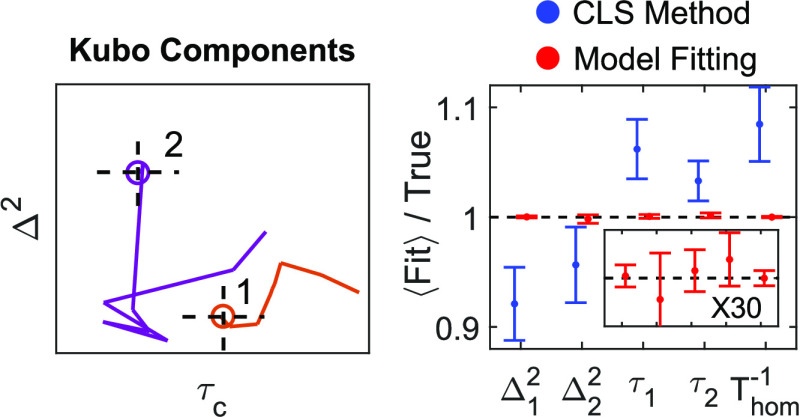

We report a comprehensive study of the efficacy of least-squares fitting of multidimensional spectra to generalized Kubo line-shape models and introduce a novel least-squares fitting metric, termed the scale invariant gradient norm (SIGN), that enables a highly reliable and versatile algorithm. The precision of dephasing parameters is between 8× and 50× better for nonlinear model fitting compared to that for the centerline-slope (CLS) method, which effectively increases data acquisition efficiency by 1–2 orders of magnitude. Whereas the CLS method requires sequential fitting of both the nonlinear and linear spectra, our model fitting algorithm only requires nonlinear spectra but accurately predicts the linear spectrum. We show an experimental example in which the CLS time constants differ by 60% for independent measurements of the same system, while the Kubo time constants differ by only 10% for model fitting. This suggests that model fitting is a far more robust method of measuring spectral diffusion than the CLS method, which is more susceptible to structured residual signals that are not removable by pure solvent subtraction. Statistical analysis of the CLS method reveals a fundamental oversight in accounting for the propagation of uncertainty by Kubo time constants in the process of fitting to the linear absorption spectrum. A standalone desktop app and source code for the least-squares fitting algorithm are freely available, with example line-shape models and data. We have written the MATLAB source code in a generic framework where users may supply custom line-shape models. Using this application, a standard desktop fits a 12-parameter generalized Kubo model to a 10^6^ data-point spectrum in a few minutes.

## 1 Introduction

The Kubo line shape is a common model of spectral diffusion in frequency fluctuation correlation functions (FFCFs) owing to its simple, closed-form expression, and flexibility to describe the limiting cases of homogeneous and inhomogeneous dephasing.^1^ As the FFCF is not a direct experimental observable, a variety of approximate metrics have been used to extract an approximate FFCF from two-dimensional (2D) frequency-resolved line shapes in two-dimensional infrared (2D IR) spectra.^2^ Some of these approaches include the nodal-line slope,^3−5^ dynamic line width,^6^ ellipticity,^7,8^ covariance,^9^ spectral phase slope,^10^ inhomogeneity index,^11^ eccentricity,^12^ centerline slope (CLS),^13^ inverse centerline slope (invCLS),^14,15^ and the correlation coefficient obtained by a 2D Gaussian fit.^16^ The most popular among these has been the CLS method, which has been used to measure spectral diffusion in a wide variety of systems, including proteins,^17−25^ lipid membranes,^26−29^ and ionic liquids.^30−38^ Relative to some other metrics, the CLS is more reliable at lower SNR, is invariant to line-shape interference from anharmonic peaks or phase twist, and is invariant to apodization time for time constants and relative amplitudes.^39^

There are, however, several shortcomings of the CLS method, which are also characteristics of most of the other common metrics. First, it is unreliable for characterizing relatively fast processes due to the short-time approximation,^13,14^ which, following the second-order cumulant expansion, ignores dephasing during coherence times. Second, it requires a second step of fitting to the linear absorbance spectrum to obtain absolute values for Kubo amplitudes and homogeneous dephasing. This fitting is problematic in situations where the linear absorption is either unavailable or unreliable due to dilute reporters, weak extinction coefficients, or spectral congestion. Furthermore, linear absorption spectra are often inaccurate near the baseline, which can distort the resulting fit parameters. Third, absolute values of Kubo amplitudes and homogeneous dephasing are sensitive to the apodization time, meaning results may vary depending on the duration of coherence time measured or how quickly the apodization filter tapers to zero.^39^ Fourth, the CLS method still requires a remarkably high SNR (e.g., ∼100:1) to yield reliable results, which has limited its application.^40^

Model fitting to the nonlinear waiting-time-dependent spectra provides a natural solution to these issues. Directly fitting the data to a user-supplied line-shape model does not depend on the short-time approximation. It allows users to match the time/frequency domain(s) in which they collect data and thereby mitigate apodization bias. It does not depend on fitting the linear absorption spectrum and therefore enables accurate measurements of spectral diffusion in a variety of scenarios that were previously inaccessible. Finally, as we will show, model fitting is reliable at far lower SNR (e.g., 10:1), is far less susceptible to structured residual signals than the CLS method, and yields reliable uncertainties for the measured line-shape parameters.

This manuscript provides a comprehensive study of how fitting generalized Kubo line shapes to multidimensional spectra compares to the CLS method in terms of the accuracy, precision, and reliability of the resulting parameters. While Garrett-Roe and co-workers have shown several examples of model fitting 2D IR waiting-time series using the fmincon function in MATLAB,^30,32,33,41^ a comparison of accuracy between model fitting and the CLS method by fitting to simulated spectra, where true values of parameters are known, has been missing. We find that our model fitting routine improves precision over the CLS method by 8–15× on average for Kubo time constants and 8–50× for Kubo amplitudes and homogeneous dephasing, which is due, in part, to a novel figure of merit used in our fitting algorithm that we refer to as the scale invariant gradient norm (SIGN). We find that numerical instabilities associated with some fitting parameters appearing nearly indistinguishable at certain points in parameter space are the primary cause of sudden ceasing, which could be mistaken for local minima. Importantly, the SIGN readily identifies these events, which enables swift correction by our fitting algorithm.

We begin the manuscript by describing our approach, including a description of the least-squares fitting program, estimation of error, the introduction of the scale invariant gradient norm, a brief review of multicollinearity (or ill-conditioning) in fitting problems, preprocessing of data prior to fitting, and description of hardware and software used in measurements and least-squares fitting. We then examine several experiments including a side-by-side comparison of model fitting and the CLS method for 100 trials of simulated data, fitting with too many or too few Kubo components, fitting to low SNR data, fitting to data with phasing errors, fitting to experimental data, and fitting to undersampled data. We then conclude with a discussion of recommended practices for model fitting.

## 2 Materials and Methods

### 2.1 Least-Squares Fitting Algorithm

The Gauss–Newton algorithm is a common approach for model fitting.^42^ Within the least-squares routine, experimentally measured data (provided as a multidimensional input) are concatenated into a one-dimensional vector **D** (*N*_D_ × 1), where information regarding dimensionality is preserved in the ordering of data. Throughout the text, we use bold letters to denote vectors and matrices. We denote the residual between data **D** and line-shape model **M**(**p**) by **r** in eq 1, where **p** (*N*_p_ × 1) is a vector of variable fitting parameters. We define “parameter” as any number subject to change with the measured system (e.g., the center frequency, homogeneous lifetime, etc.), while the preceding adjectives “constant” or “variable” refer to the status of a parameter during least-squares fitting. For generalized least-squares, the cost function *C*(**p**) (a.k.a. χ^2^) is equal to the quadratic form in eq 2, where **V**_**D**_ (*N*_D_ × *N*_D_) is proportional to the data variance–covariance matrix,^43^ and superscript **T** denotes the transpose. In the simplest case of uniform and uncorrelated noise, **V**_**D**_ is equal to the identity matrix and the cost function *C*(**p**) = **|r|**^**2**^. For more complicated cases of noise, a detailed discussion of **V**_**D**_ is provided in Section 2.2
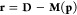
1
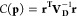
2The objective of least-squares fitting is to minimize eq 2 subject to **p**. Because **r** depends nonlinearly on **p**, minimizing the cost function C(**p**) requires an iterative process: **p**_**i**+**1**_ = **p**_**i**_ + **Δp**. At each iteration, the second-order Taylor series shown in eq 3 locally approximates *C*(**p**), where ∇**C** (1 × *N*_p_) and ∇∇**C** (*N*_p_ × *N*_p_) are the gradient and Hessian of *C*(**p**), respectively, with respect to **p**
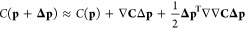
3Applying the gradient ∇ to eq 2, we find a more useful expression for ∇**C** in eq 4 given in terms of **r**, **V**_**D**_, and the Jacobian **J** (*N*_D_ × *N*_p_), which is a matrix composed of partial derivatives ∂**M**/∂p_k_ computed by finite-difference approximation
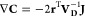
4Equation 5 provides an expression for ∇∇**C** in terms of the residual-weighted Hessian of the model (**H**_j,k_ = **r**^T^**V**_D_^–1^∂^2^**M**/∂p_j_∂p_k_). Simply put, the most salient difference between several popular algorithms is in how they compute ∇∇**C**. Newton’s algorithm computes it exactly as ∇∇**C** = 2(**J**^T^**V**_D_^–1^**J** – **H**).^42^ Gauss–Newton computes it approximately as ∇∇**C** = 2**J^T^V**_D_^–1^**J**. Levenberg–Marquardt computes a more stable approximation ∇**C** = 2(**J^T^V**_D_^–1^**J** + λ)^42,44,45^ or some variation thereof,^46^ where λ is known as the damping parameter and  is the identity matrix. Finally, steepest descent simply assumes ∇∇**C** = .^42^ The advantage of the Gauss–Newton algorithm over Newton is the time saved in not computing **H**, which is usually quite significant. Newton is also susceptible to convergence problems far from the global minimum, unlike the other algorithms mentioned. The Gauss–Newton approximation is often justified because near the global minimum the residual **r** is relatively small and mostly random with zero mean, implying **H** is negligible. The Levenberg–Marquardt algorithm interpolates between the limiting cases of Gauss–Newton (λ → 0) and steepest descent (λ→∞). The advantage of Levenberg–Marquardt is the added stability of inverting ∇∇**C** due to the λ term; however, this is irrelevant in our case as our algorithm guards against singular ∇∇**C** (Section 2.3). The disadvantage of Levenberg–Marquardt is that successful optimization for λ is difficult to predict and usually requires a dynamic routine. Furthermore, as λ increases, Levenberg–Marquardt behaves more like steepest descent, which is slower to converge near minima because ∇∇**C** → λ ignores the true curvature of *C*(**p**). Therefore, we have chosen the Gauss–Newton approximation in eq 5
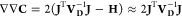
5The minimum of eq 3, Δ**p**, is obtained using the MATLAB syntax **Δp** = −∇∇**C**\∇**C**^**T**^, where “\” corresponds to the MATLAB function mldivide, which solves the linear system of equations in eq 6. Note that ∇∇**C** may not be invertible on occasion. As is the standard practice with nonlinear fitting routines, Δ**p** undergoes a quality check at the end of each iteration to ensure the move is productive. In particular, the program uses a backtracking line search subject to the Armijo condition. And finally, the program compares the new position **p**_**i**+**1**_ = **p**_**i**_ + **Δp** to the parameter boundaries provided by the user and corrects Δ**p** if necessary
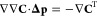
6Occasions may arise in which the solution for Δ**p** in eq 6 is inaccurate or unacceptable in a directional sense. Consequently, iterative changes in Δ**p** approach zero even though ∇**C** is clearly nonzero. We refer to this as algorithmic stalling. Stalling is a separate issue from a local minimum in that ∇**C** = **0** in a local minimum. We find that simply sending **p** to a random point within user-supplied boundaries, which we refer to as a random restart, is a reliable strategy for resolving a stall. This approach is closely related to multistart,^47^ which is a shotgun strategy for problems plagued by local minima.

### 2.2 Uncertainty of Fit and Nonuniform Noise

The parameter variance–covariance matrix **V**_**p**_ (*N*_p_ × *N*_p_) given by eq 7 provides the uncertainty of the variable fitting parameters assuming that **p** is located at the global minimum.^43^ We provide a derivation of eq 7 in Supporting Information Section D
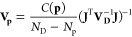
7Note that eq 7 assumes that **V**_**D**_ (*N*_D_ × *N*_D_) is proportional to the data variance–covariance matrix. If noise is uncorrelated across **D**, but not necessarily uniform, then **V**_**D**_ is a diagonal matrix with elements proportional to the variance of each datum. For example, if datum *i* is averaged twice as much as datum *k*, then **V**_D*_ii_*_^–1^/**V**_D*_kk_*_^–1^ = 2/1 and hence the convenience of referring to **V**_D_^–1^ instead of **V**_**D**_. We emphasize that **V**_**D**_ need only be *proportional* to the true variance–covariance matrix in eq 7 since *C*(**p**) ∝ **V**_D_^–1^ and **(J^T^V**_D_^–1^**J)**^–1^ ∝ **V**_**D**_. If noise is both uncorrelated and uniform across **D**, then **V**_D_^–1^ is the identity matrix.

Accounting for correlated noise more generally is challenging because the nondiagonal data variance–covariance matrix **V**_**D**_ of size *N*_D_ × *N*_D_ might easily occupy a terabyte of memory for multidimensional spectra. Hence, for feasibility sake, our program assumes that **V**_**D**_ is diagonal, which is typical for most other fitting programs. Consequently, the fitting algorithm is most optimal for spectra with uncorrelated noise. While conventional referencing schemes are unreliable for achieving uncorrelated noise, *calibrated* referencing schemes are known to achieve virtually uncorrelated noise,^48−50^ which can also be realized using 100 kHz Yb laser systems.^51−53^ Nevertheless, Supporting Information Section F provides a comparison of model fitting to edge-pixel referenced^48^ and unreferenced data, which suggests that model fitting to unreferenced data is still reliable, setting aside the expected gain in uncertainty from the larger noise.

### Scale Invariant Gradient Norm 

2.3

Stopping criteria are a notorious complication with fitting algorithms. They are often based on user-specified thresholds. Three common examples are |Δ**p**| < 10^–4^, |*C*(p_i+1_) – *C*(p_i_)| < 10^–5^, or **|**∇**C|** < 10^–6^. The threshold values of 10^–4^, 10^–5^, and 10^–6^ in these examples are arbitrary and may strongly depend on factors such as the scaling of parameters |**p**|, data |**D**|, noise |**V**_**D**_|, and number of data points *N*_D_. Therefore, users must reconsider these thresholds on a case-by-case basis, often empirically. To that end, we propose a new stopping criterion, which we refer to as the scale invariant gradient norm, , defined in eq 8. We motivate this expression by unit analysis of **|∇C|**: *C*(**p**) cancels with the numerator of ∇**C**, and each element of  cancels with a corresponding element of ∂p_i_ in the denominator of ∇**C**. In eq 8, the numerator is evaluated at iteration *i*, where ∇**C** is of size 1 × *N*_p_ (eq 4) and  is of size *N*_p_ × 1 (the diagonal root of eq 7), while *C*(**p**) (a scalar, eq 2) in the denominator is evaluated at iteration *i* – 1 (evaluating at iteration *i* - 1 is better behaved during random restarts)
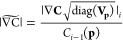
8In addition to serving as a stopping criterion,  reliably indicates when the algorithm is stalled, which is resolved by random restart. Our stopping criteria require that the previous three iterations have  < 10^–9^ and have *C*(**p**) within 10% of the lowest *C*(**p**) encountered in all previous iterations, which provides some moderate protection against local minima. Regardless, we show that local minima are virtually nonexistent for three-level systems, so the second stopping criterion is somewhat moot. If an iteration does not meet stopping criteria, then the program checks for a stall. Our stalling criteria require that the last three iterations have less than 1% deviation in  and less than 1% deviation in *C*(**p**). The program also triggers a stall if ∇∇**C** is singular or nearly singular. If no stall is detected, then the program continues to the next iteration.

### 2.4 Multicollinearity Considerations

Considerations of multicollinearity, also known as ill-conditioning, are *essential* to developing a successful fitting model. We speculate that the limited application of multidimensional fitting algorithms in nonlinear spectroscopy to date is due, in part, to overlooking this aspect of models. A fitting problem is said to be multicollinear (or ill-conditioned) if column vectors of the Jacobian **J** are nearly linearly dependent, which causes instability in computing inverse matrices associated with **J**, such as the Hessian ∇∇**C**^–1^ and **V**_**p**_.^54,55^ Consequently, the calculation of Δ**p** in eq 6 may be inaccurate or unstable (i.e., a major cause of stalling) and covariances among those nearly linearly dependent parameters become overwhelming. In more extreme cases of multicollinearity, tiny perturbations of noise cause wild fluctuations in fitting parameters,^56,57^ and error estimates become useless.^54,56,58^

Not surprisingly, parameter redundancy, or indistinguishability between parameters, drives multicollinearity^59^ and should be avoided whenever possible. Here, we list a few examples of multicollinearity.

Example 1: homogeneous dephasing is a limiting case of a Kubo line shape, where *T*_Hom_^–1^ approaches the product Δ^2^τ. In this case, Δ^2^ and τ are indistinguishable. Hence, homogeneous dephasing is modeled by a single fitting variable, *T*_Hom_^–1^.

Example 2: we refer to a pair of Kubo components with similar correlation times (τ_1_ ≈ τ_2_) but different amplitudes (Δ_1_^2^ ≠ Δ_2_^2^) as degenerate. In this case, Δ_1_^2^ and Δ_2_^2^ become indistinguishable due to their linear dependence in the FFCF, corresponding to Δ_1_^2^ exp(−*t*/τ_1_) + Δ_2_^2^ exp(−*t*/τ_2_) ≈ (Δ_1_^2^ + Δ_2_^2^) exp(−*t*/τ_1_).

Example 3: when modeling an isotropic response, the total homogeneous dephasing (*T*_Hom_) has three contributions from pure dephasing (*T*_2_^*^), vibrational lifetime (*T*_LT_), and orientational relaxation (*T*_or_): 1/*T*_Hom_ = 1/*T*_2_^*^ + 1/2*T*_LT_ + 1/3*T*_or_. If the waiting-time axis is sufficiently well sampled to ensure linear independence of *T*_LT_, the remaining three lifetimes *T*_Hom_, *T*_2_^*^, and *T*_or_ are still multicollinear with one another. This is equivalent to trying to solve an algebraic equation for the values of *x*, *y*, and *z* given only that *x* = *y* + 5/2 + *z*/3. There are infinite possible answers. So, we reduce the number of unknowns to simply *T*_Hom_ and *T*_LT_, without specifying *T*_2_^*^ and *T*_or_ while fitting the isotropic response but enforce the boundary condition 1/*T*_Hom_ > 1/2*T*_LT_.

Example 4: due to calibration error, the 0–1 peak may slightly differ in location on the pump and probe axes (e.g., <1 cm^–1^). Therefore, it is okay to model a calibration error along one of the axes, but modeling it along both axes, or modeling two calibration errors (one for each axis), would cause indistinguishability between the calibration error(s), the 0–1 center frequency, and the anharmonic shift.

A common measure of multicollinearity is the variance inflation factor (VIF).^55,60^ As the name suggests, the VIF is the factor by which the variance of a parameter inflates due to collinearity with other variable parameters. The VIF of the *i*th parameter may be computed empirically by measuring the variance over many simulated trials for two scenarios: (1) one in which all fitting parameters are varied during each fit (as is usual) and (2) only the *i*th parameter is varied during the fit and all other fitting parameters are held constant at their true values. Then, the VIF is the ratio of the former to the latter. However, the empirical method may be time consuming, so we elect for the equivalent theoretical expression: column vectors of the Jacobian **J** are normalized to **Ĵ** such that all diagonal elements of **Ĵ**^**T**^**V**_D_^–1^**Ĵ** are equal to one. Then, the VIF of the *i*th parameter is equal to the *i*th diagonal element of (**Ĵ**^**T**^**V**_D_^–1^**Ĵ**)^−1^.^55,60^ To understand this, consider the limiting case of perfectly orthogonal model parameters and uniform, uncorrelated noise (i.e., **V**_**D**_ is the identity matrix), then **Ĵ**^**T**^**V**_D_^–1^**Ĵ** is the identity matrix, and hence, VIF is equal to 1 for every parameter. In the other limiting case in which any two or more parameters are perfectly linearly dependent, then **Ĵ**^**T**^**V**_D_^–1^**Ĵ** is rank-deficient, causing det(**Ĵ**^**T**^**V**_D_^–1^**Ĵ**) = 0, and the diagonal of (**Ĵ**^**T**^**V**_D_^–1^**Ĵ**)^−1^ blows up to infinity. For that reason, we recommend the true matrix inverse for testing VIF, not a pseudo-inverse.

### 2.5 Preprocessing Prior to Fitting

We prefer to fit data in the original measurement domain, which is (τ_1_, *T*_w_, ω_3_). Therefore, our model requires a fast Fourier transform (FFT) along the probe axis. The FFT assumes equal spacing along the probe axis, but, due to the spectrograph, our experimental data points are nonlinearly spaced along the probe axis. Therefore, to match the data and model in eq 1, users should preprocess the data by interpolating along a linear probe axis. This is a straightforward task using the spline function in MATLAB. The GUI version of our program does this automatically when loading data.

Time domain data are commonly padded by an equal number of zeros just prior to the Fourier transform to enforce causality, which we call causal zero padding. We refer to zero padding beyond this point as superfluous, but it is a common practice to interpolate the data in the frequency domain. More precisely, this approach results in sinc interpolation in the frequency domain. Sinc ringing is nearly always mitigated by choosing a nonrectangular windowing function to make the data smoothly taper to zero prior to zero padding.^61^

Both superfluous zero padding and apodization manipulate data in different ways, and these perturbations will propagate into the fitting results. In fact, assuming that the true parameters are known, the change in fitting parameters **dp** due to a perturbation in data **dD** is computed by eq S10. Therefore, we generally recommend fitting data in the original measurement domain without causal or superfluous zero padding or apodization. When this is not an option, the data should be transformed back into the original measurement domain and the nonrectangular apodization window should be inverted to retrieve the original data. Careful consideration is needed when constructing the inverse window function (1) to avoid dividing by zero or near-divide by zero and (2) whether the original filter had 1/2 scaling of the DC component, which is common practice.^61,62^

Data are collected using a pulse shaper,^63,64^ which ensures accurate phasing of all 2D IR spectra, though we have added an optional fitting parameter to account for a uniform zero-order phasing error across all spectra. Results of model fitting to simulated data with phasing errors are provided in Supporting Information Section G.

### 2.6 Computer, Software, and Computational Time

We use a standard laptop to run model fitting and data analysis in MATLAB R2020a, i.e., an Intel Core i7-8550U CPU @ 1.80GHz, 16 GB of RAM, and (optionally) an NVIDIA GeForce GTX 1050 GPU, 4 GB GDDR5. The standalone desktop app (available for Windows and Mac users), MATLAB source code, and experimental data are freely available at https://github.com/kevin-robben/model-fitting. Detailed instructions for reproducing all data and analyses are provided in Section H of the Supporting Information.

The time needed to fit a single waiting-time series is approximately equal to ζ × *N*_D_ × *N*_p_, where ζ is a constant specific to the computer, *N*_D_ is the number of data points, and *N*_p_ is the number of variable fitting parameters. The Jacobian **J** requires 2*N*_p_ unique calculations of the model **M**(**p**) for the central finite-difference approximation, and hence, the bottleneck of the program is computing **M**(**p**). We estimate ζ ≈ 1.3 × 10^–7^ min/point/parameter for CPU computing on the laptop described above. For spectra smaller than 10^6^ points, ζ is roughly the same between CPU and GPU computing. For spectra larger than 2 × 10^6^ points, GPU computing reduces ζ by a factor of 2 or greater.

Aside from GPU computing, the computational time is reduced by fitting to fewer data points, which may be achieved by averaging more laser shots with the minimum necessary data points. For a simple three-level system, the pump axis can be shortened to, e.g., 16 data points with careful consideration of the rotating frame. This may be achieved by measuring just 16 points along the pump axis with, e.g., 250 fs steps or, alternatively, by deleting excess data points along the pump axis in the frequency domain that fall outside the region of interest and then transforming back to the time domain. However, as mentioned in Section 2.5, careful consideration of the window function is needed when switching between time and frequency domains.

### 2.7 Linear and 2D IR Measurements

We collect FTIR measurements on a Bruker Tensor 27 with a 1 cm^–1^ resolution. 2D IR measurements are collected at 2 kHz with ∼150 fs pulses centered at ∼2150 cm^–1^ and magic-angle polarization.^48^ The 2020 data are collected with 15 μJ pump energy and 200 mM MeSCN in DMSO. The 2021 data are collected with 2 μJ pump energy and 400 mM MeSCN in DMSO. Edge-pixel referencing subtracts the correlated local-oscillator noise.^48^ The 2020 data are collected with a 4-pulse, real-valued phase cycle, while the 2021 data are collected with an 8-pulse, complex-valued phase cycle. Comparative tests have led us to conclude that model fitting works equally well for fitting to real-valued or complex-valued free induction decays, given an equal number of laser shots.

### 2.8 Line-Shape Models

Data are modeled as the isotropic response of a three-level system with , where the first term accounts for homogeneous dephasing and the summation accounts for multiple Kubo components when applicable. Homogeneous dephasing of the 1–2 transition is modified during the second coherence time to account for lifetime broadening of the 1–2 transition.^65^ Further details and complete equations are provided in Supporting Information Section C.

## 3 Results and Discussion

### 3.1 Fitting Simulated Data

First, we test the accuracy and precision of the algorithm by model fitting to simulated data and comparing the fit parameters to known, true values. We simulate the isotropic response of the C≡N stretch of MeSCN in H_2_O, as characterized by Yuan and Fayer.^66−68^ We add Gaussian noise (SNR of ∼ 600:1) to the free induction decay to simulate experimental data. Here, we define signal in the SNR calculation as the peak magnitude of the 0–1 transition of the transient absorption spectrum at zero waiting time. Our choice to simulate data with 600:1 SNR avoids occasional unphysical results that would otherwise occur in the CLS analysis of data with lower SNR over the course of 100 trials. Nevertheless, we also show that model fitting can be reliable for data with an SNR of 10:1 (vide infra) subject to the expected increase in variance predicted by eq 7.

The variable fitting parameters comprising **p** are as follows: (1) the 0–1 peak amplitude A_01_, (2) the 1–2 peak amplitude A_12_, (3) the 0–1 center frequency ω_01_, (4) a calibration mismatch error between the pump and probe axes δω_1_, (5) the anharmonicity Δ_Anh_, (6) the first Kubo time constant τ_1_, (7) squared amplitude Δ_1_^2^, (8) the second Kubo time constant τ_2_, (9) squared amplitude Δ_2_^2^, (10) a scaling factor for the Kubo amplitude of the 1–2 transition relative to the 0–1 β, i.e., Δ_1_^2^(1–2) = β^2^Δ_1_^2^(0–1) and Δ_2_^2^(1–2) = β^2^Δ_2_^2^(0–1), (11) the inverse vibrational lifetime*T*_LT_^–1^, and (12) the inverse homogeneous lifetime *T*_Hom_^–1^. The calibration mismatch error δω_1_ effectively acts like a zero-order frequency shift along the pump axis, which accounts for a finite error in the independent calibrations of the pump and probe axes. Though calibration errors are not present in the simulated data, we still treat the fitting as we would with experimental data, where this parameter may be necessary. δω_1_ is also useful for fitting to phase-distorted data, as shown in Supporting Information Section G. The Kubo amplitude scaling factor β may partially account for situations where dephasing does not scale harmonically between the 0–1 and 1–2 transitions. We tend to find that fitting to inverse lifetimes and squared amplitudes, i.e., *T*_Hom_^–1^ and Δ^2^, is more stable than *T*_Hom_ and Δ, which is not surprising given that *T*_Hom_^–1^ and Δ^2^ appear linearly in exponential arguments of the response function model. At the end of every iteration, the program checks to ensure that the next movement **p + Δp** is within user-defined boundaries. This routine could be modified to ensure that *T*_Hom_^–1^ > 1/2 *T*_LT_^–1^ + 1/3 *T*_or_^–1^, which is a physical requirement.^14^ For the present cases of isotropic polarization, however, *T*_or_ is not known a priori, so we settle for *T*_Hom_^–1^ > 1/2 *T*_LT_^–1^ in our boundary checks. Usually, 1/3 *T*_or_^–1^ does not contribute much to *T*_Hom_^–1^, so we think this is a reasonable approximation for convenience, though model fitting to polarization-dependent spectra to fit *T*_or_ has been done.^32^

For comparison, we also analyze the simulated data using the centerline-slope (CLS) method.^13,14^ One feature of 2D Kubo line shapes is that they are asymmetric in frequency and the asymmetry is, itself, frequency-dependent (see Figure S1). This effect leads to inaccurate centerline measurements when fitting the slices with a symmetric function. Therefore, we fit an asymmetric Lorentzian (Lorentzian + linear term + offset) to slices along the probe axis and then measure the peak of the asymmetric fitting function by numerically finding the extremum of the interpolated function to obtain the centerline points. This method provides a more accurate measure of the true centerline and perfectly agrees with Falvo’s analytical expression for calculating the CLS decay by double integration of the response function^69^ when tested on our simulated spectra. Fitting a biexponential model to the CLS decay yields the Kubo time constants. For this analysis, we include all waiting-time points, including *T*_W_ = 0. We measure homogeneous dephasing and Kubo amplitudes by fitting to the upper 80% of the linear absorption spectrum, holding the Kubo decay times constant. Many baseline distortions are known to occur in FTIR measurements including interference fringes, atmospheric absorption, and scattering from scratched windows, particles, and aggregates.^70^ Any subtle variation between background and sample measurements, such as path length, index of refraction, concentration and location of particles or aggregates, window scratches, temperature, pressure, atmosphere, and other conditions, can lead to residual distortions in background-subtracted FTIR spectra. These distortions, in addition to various forms of noise, are nontrivial to model, so we do not include them in our simulation. In fact, to consider the best-case scenario for the CLS, we fit to a noiseless simulation of the linear absorption spectrum. Nevertheless, we note that in real samples, these contributions would all lead to even greater uncertainty for the line-shape parameters from the CLS method.

**Figure 1:**
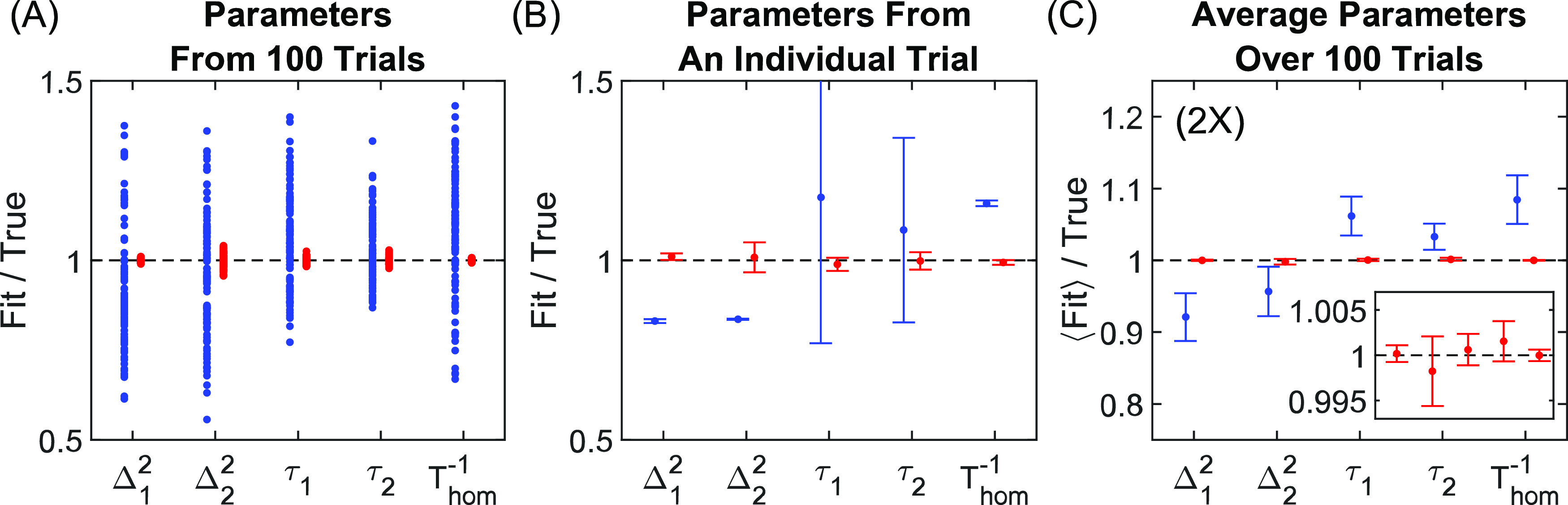
Dephasing parameters by CLS method (blue) and model fitting (red) of simulated 2D IR waiting-time series. (A) Fit parameters from all 100 trials. (B) Fit parameters obtained from an individual trial (chosen at random) with 95% confidence intervals estimated from the covariance of fit. (C) Average of fit parameters over all 100 trials with 95% confidence interval calculated from the standard error of the mean, with the inset showing a zoom-in of the true-value line.

Figure 1 shows side-by-side comparisons of the line-shape parameters from both the CLS method (blue) and model fitting (red). For each trial, the code generates a new sampling of random noise for the spectra and chooses a random starting point for fitting **p**. Each panel shows, from left to right, the line-shape parameters: the squared amplitude of the first and second Kubo components (Δ_1_^2^ and Δ_2_^2^), the correlation time of the first and second Kubo components (τ_1_ and τ_2_), and the inverse homogeneous lifetime (T_Hom_^–1^). Panel (A) shows the results of all 100 trials plotted as the ratio of fit to true value. Video S1 shows fits to each of the CLS decays for all 100 trials.

Panel (B) shows the results for an individual trial (selected at random) with 95% confidence intervals derived from the covariance of the fit in eq 7. Corresponding plots of panel (B) for every single trial (Video S2) confirm that, for the model fitting approach, the true values fall within the confidence intervals for ∼95% of trials. In contrast, the CLS method grossly underestimates the confidence intervals for the Kubo amplitudes and homogeneous dephasing. In calculating these confidence intervals for the CLS method, it was assumed that the Kubo time constants were known with absolute certainty, which is currently the standard treatment. In reality, the Kubo time constants obtained from CLS fits are uncertain, with reported errors ranging from 5 to 50%.^66,67,71,72^ This additional uncertainty must be accounted for when fitting the linear absorption spectrum if we hope to achieve accurate uncertainties for homogeneous dephasing and Kubo amplitudes. Accounting for this effect involves propagating the uncertainty of the Kubo time constants through the standard variance–covariance matrix of the linear absorption fit to obtain the modified variance–covariance matrix (eq S14), which we derive in Supporting Information Section E. To the best of our knowledge, CLS error bars have never been reported using eq S14.

Panel (C) shows the average values and 95% confidence intervals over 100 trials, where model fitting improves precision over the CLS method by 8–15× for Kubo time constants and 8–50× for Kubo amplitudes and homogeneous dephasing. That true values fall within the intervals in panel (C) suggests that the model fitting is highly accurate and reliable when provided an appropriate model for the data. On the other hand, the CLS method is inaccurate by 5–10% on average.

**Figure 2:**
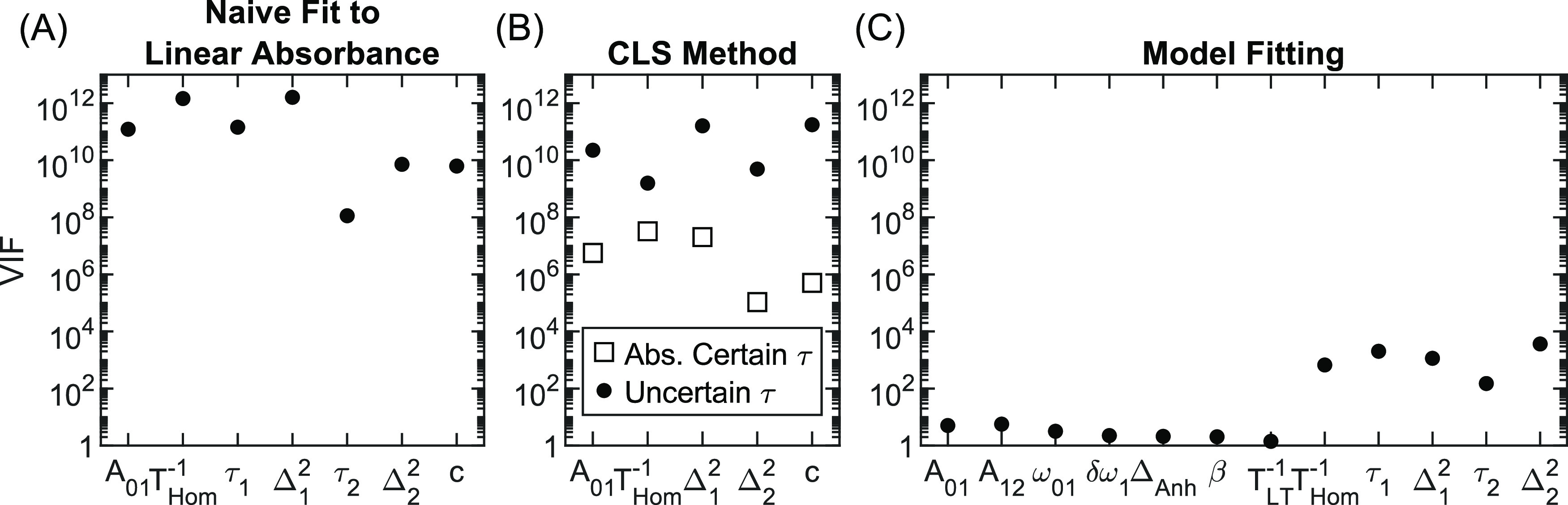
Variance inflation factor (VIF) is a measure of multicollinearity (or ill-conditioning) in least-squares regression. Plots of VIFs for (A) naively fitting all dephasing parameters (with scaling and offset) to a linear absorption spectrum, (B) the CLS method, and (C) model fitting. Larger VIFs reflect higher multicollinearity.

Propagation of error difficulties aside, fitting dephasing parameters to the linear absorption spectrum is severely ill-conditioned even with the constraints afforded by the CLS method. Figure 2 shows VIFs (see Section 2.4) for three different scenarios. Panel (A) is for naively fitting to the upper 80% of the linear absorbance spectrum with a linear response model that includes an amplitude (A_01_), all five dephasing parameters (*T*_Hom_^–1^, τ_1_, Δ_1_^2^, τ_2_, Δ_2_^2^), and a constant offset (c), which is a scenario that is well known to yield poorly constrained fitting parameters. The resulting VIFs range between 10^8^ and 10^12^. As an example, consider the result of Δ_1_^2^ with a VIF of 10^12^, which is the inflation that is expected in the variance of Δ_1_^2^ because one, or more, covariances exist between Δ_1_^2^ and other fitting variables. If the other six parameters were held constant while fitting Δ_1_^2^, then the uncertainty of Δ_1_^2^ should decrease by a factor of 1 000 000 (i.e., ). In this scenario, the uncertainty of every parameter is so large that any tiny perturbation in the noise causes massive fluctuations in values of the fitting parameters. These results clearly demonstrate that naively fitting all dephasing parameters to the linear absorbance spectrum is an ill-conditioned problem, consistent with prior expectations.^61,73^

Panel (B) shows results for fitting to the upper 80% of the linear absorbance spectrum using constraints on the time constants similar to that done in the CLS method. Here, we consider the cases where the Kubo time constants are known with absolute certainty (squares) or 10% uncertainty (circles). For the more realistic case of uncertain time constants, VIFs range between 10^9^ and ∼10^11^, which imply roughly ∼100 000× inflation in the uncertainties of Kubo amplitudes and homogeneous dephasing, give, or take an order of magnitude. This result explains the large variance seen in panel (A) of Figure 1 and the necessity of simulating data with a 600:1 SNR in the 2D IR spectra to obtain reliable results for the CLS method. VIFs differ by 4–5 orders of magnitude between absolutely certain and uncertain Kubo time constants, which implies that error bars on Kubo amplitudes and homogeneous dephasing are underestimated by 2 orders of magnitude. Indeed, this result is observed for the CLS method in Figure 1B and Video S2. Finally, panel (C) shows VIFs for model fitting to the 2D IR waiting-time series. For nondephasing parameters, including the vibrational lifetime, all VIFs are less than 10, meaning multicollinearity is negligible for these parameters. On the other hand, VIFs range between 100 and 3000 for the remaining dephasing parameters, implying inflated uncertainties between 30× and 50×. Though significant, the inflation of uncertainties in Kubo amplitudes and homogeneous dephasing remains 2–4 orders of magnitude smaller than for the CLS method.

**Figure 3:**
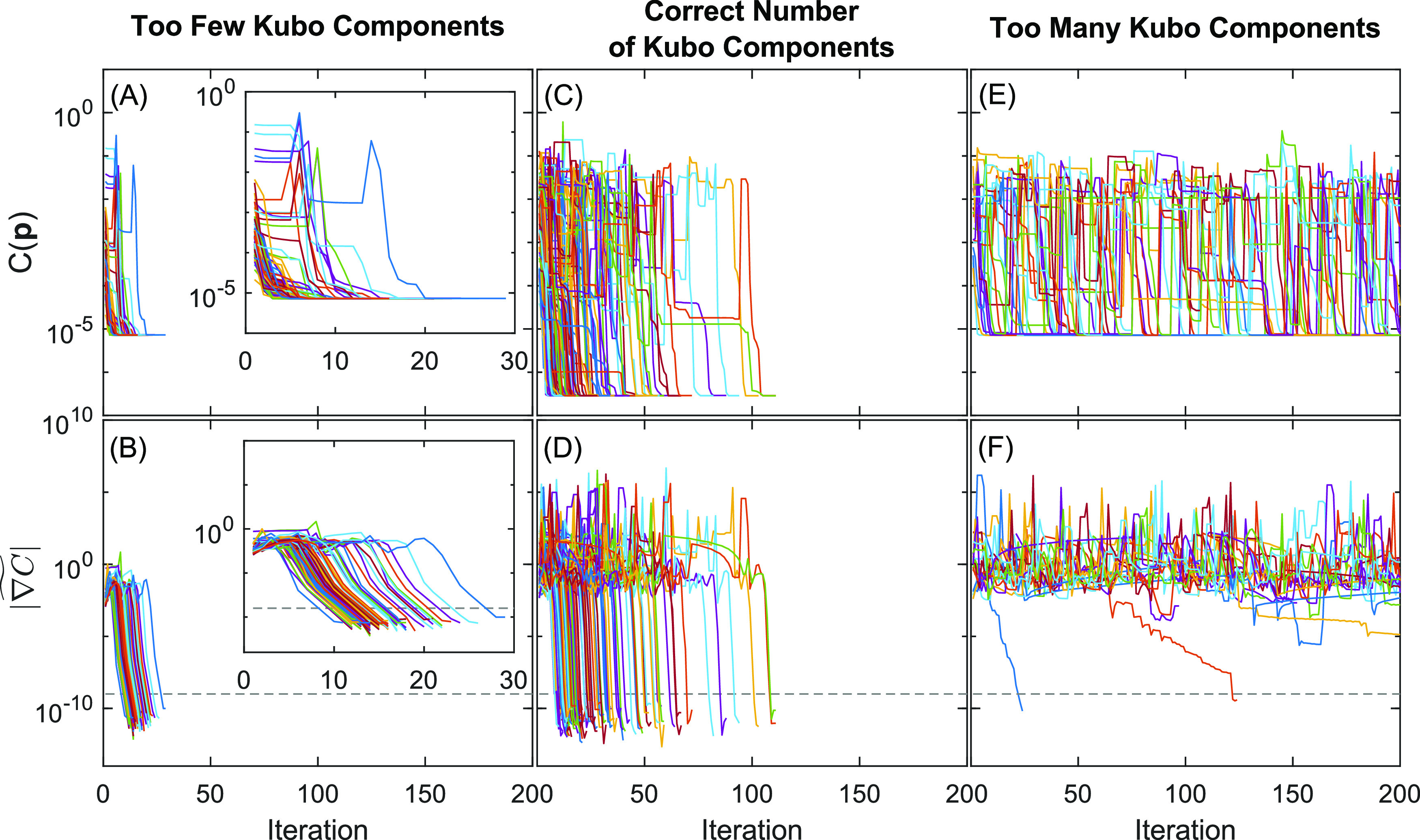
Plots of the cost function and SIGN for modeling with too few (A, B), the correct number (C, D), and too many (E, F) Kubo components. Each trial corresponds to a random sampling of noise and random starting point for **p**. The dashed line is associated with the stopping criterion.

In addition to comparing model fitting to the CLS method, we are also interested in evaluating the robustness of the model fitting approach to variations in the chosen model, which may not always be known a priori. Figure 3 shows plots of *C*(**p**) and  versus fitting iteration for three scenarios in which the fitting model **M**(**p**) has one less (left), the same number (middle), and one more (right) Kubo component(s) than are actually present in the simulated data **D**. We start with the analysis of the middle panels (C) and (D), which correspond to the fitting results shown in Figure 1. Plots of *C*(**p**) in panel (C) show that *C*(**p**) is useful for broadly comparing the quality of fit for a given iteration of **p** but is not particularly useful for understanding convergence because progress near the global minimum is difficult to evaluate on a relative scale. On the other hand,  shows simple and reproducible behavior that clearly indicates convergence. The variation of  with iteration shows three phases. First, when **p** is far away from a minimum of the cost function, the gradient is ∼10^–1^. Next, **p** reaches the neighborhood of a minimum where the second-order approximation of *C*(**p**) in eq 3 is highly accurate, and hence,  rapidly descends many orders of magnitude in as little as 2–3 iterations. Finally, **p** reaches the minimum of *C*(**p**) but  approaches an asymptote. This asymptote should be essentially invariant at the global minimum because  is, effectively, the relative roundoff error in *C*(**p**) and hence is determined by the accumulated roundoff errors in eq 8 on top of machine precision (∼2 × 10^–16^). In a few cases,  descends halfway and stalls. The algorithm responds to stalling by restarting from a new randomly generated **p** within the user-supplied boundaries corresponding to the sudden increases seen in both plots (C) and (D). The dashed line in panel (C) is a user-defined limit associated with the stopping criteria (Section 2.3). Video S3 shows fitting trajectories for every trial.

Panels (A) and (B) show plots of *C*(**p**) and  for 100 trials of fitting a one-Kubo model to data simulated by a two-Kubo line shape. Compared to panels (C) and (D), fitting is far less susceptible to stalling and converges significantly faster, which suggests that *C*(**p**) is more convex over a wider range of **p**. Past a point of  ≈ 10^–7^, the second-order approximation of *C*(**p**) is less effective and converges at a moderately slower rate. Fitting trajectories in Video S4 show that **p** consistently reaches the same global minimum for all 100 trials.

Panels (E) and (F) show plots of *C*(**p**) and  for 20 trials of fitting a two-Kubo model to data simulated by a one-Kubo line shape. The many jumps in both plots are evidence of frequent stalling and random restarts, which is the opposite of the behavior seen in panels (A) and (B). We ran this experiment for only 20 trials due to the large number of iterations, the resulting crowding in the plots, and the larger memory requirement for the video.

**Figure 4:**
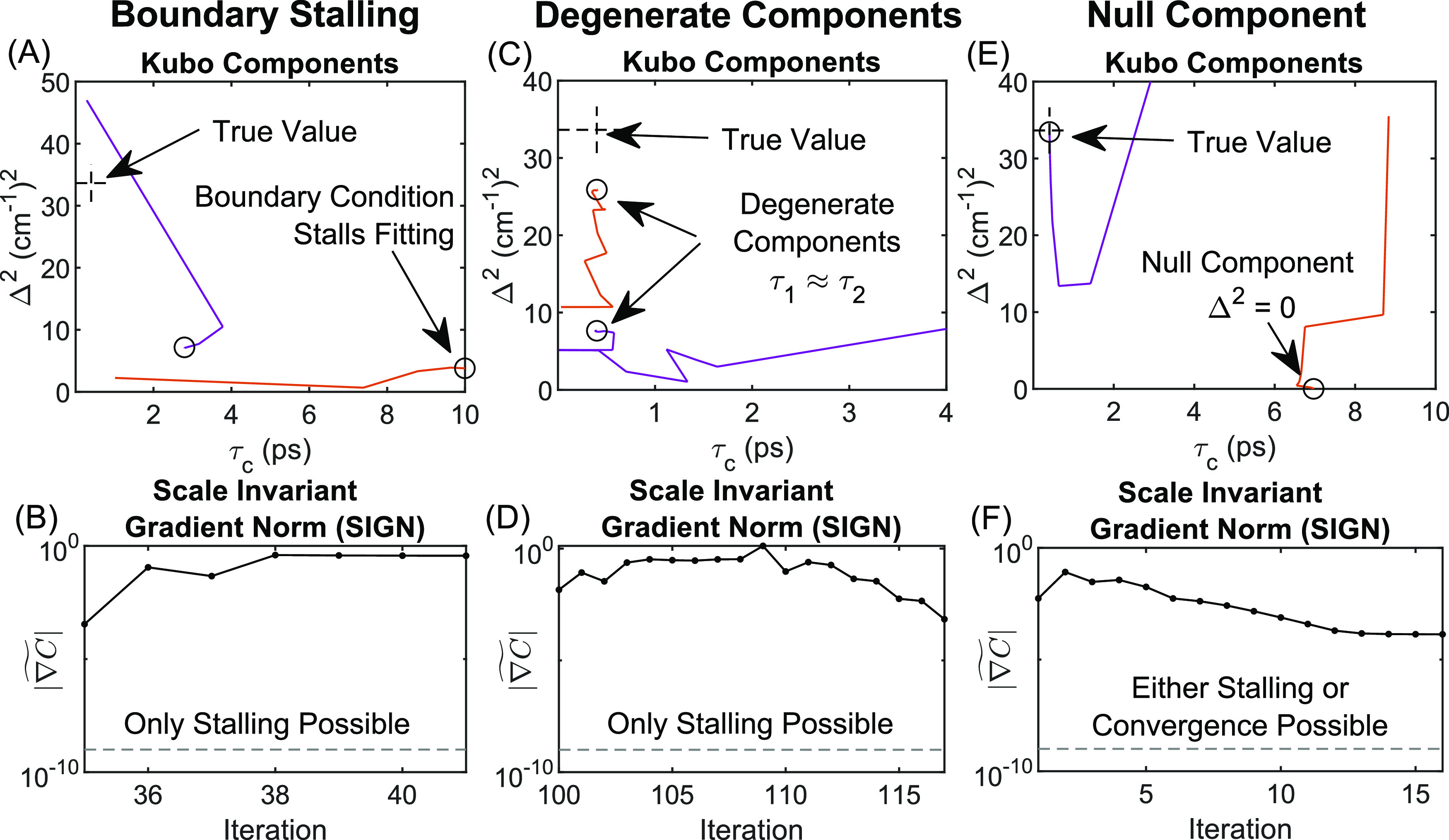
Select examples of stalling or unusual convergence. (A, B) An example of boundary stalling. (C, D) An example of stalling due to multicollinearity between degenerate Kubo components. (E, F) An example in which one component approaches the true value and the other approaches null (i.e., Δ^2^ = 0), where either stalling or convergence may occur.

Fitting trajectories in Video S5 show many examples of stalling and abnormal convergence corresponding to panels (E) and (F) in Figure 3. Stalling occurs when a single fitting iteration, ending with |Δ**p|** ≈ 0, starts an insidious cycle where **p** does not change and so the algorithm is doomed to repeat itself barring some intervention. This is distinct from local or global minima because stalling also requires  > 10^–9^, which implies that **p** is not a minimum of C(**p**). Two scenarios for which stalling may occur are (1) Δ**p** is accurate in direction but |Δ**p|** is limited by a boundary condition, which causes |Δ**p|** ≈ 0 or (2) multicollinearity among fitting parameters leads to a nearly singular ∇∇**C**, which results in an erroneous vector direction of Δ**p** (eq 6) and hence Δ**p** is unable to reduce the cost function and so |Δ**p|** ≈ 0. Figure 4 illustrates three such examples of stalling. For each example, the top panel (A), (C), or (E) shows fitting trajectories of Kubo amplitudes (*y*-axis) versus time constants (x-axis), where the dashed line reticles mark the “true” value from the simulation input, and the bottom panel (B), (D), or (F) shows the plot of SIGN versus iteration for the fit. The first example in the left panel of Figure 4 shows the case of boundary stalling, in which the direction Δ**p** is accurate for further minimizing the cost function, but the location **p + Δp** is outside of the user-defined boundary for at least one of the parameters (in this case, τ_c_ ≤ 10). Convergence ( ≪ 1) is impossible in the case of regular boundary stalling, and the program readily detects the constant value of  and resolves the stall with a random restart.

The second example in the center panel shows the formation of a degenerate pair of Kubo components (i.e., having the same time constant). Plots of VIFs shown in Videos S3 and S5 are perfect examples of multicollinearity when a degenerate pair aligns. When a degenerate pair aligns (τ_1_ ≈ τ_2_), the FFCF reduces to Δ_1_^2^ exp(−*t*/τ_1_) + Δ_2_^2^ exp(−*t*/τ_2_) ≈ (Δ_1_^2^ + Δ_2_^2^) exp(−*t*/τ_1_) and hence the amplitudes Δ_1_^2^ and Δ_2_^2^ are linearly dependent and therefore indistinguishable. As shown in many examples of Videos S3 (e.g., trial 5, iteration 26) and S5 (e.g., trial 3, iteration 111), the VIFs and condition number always explode to infinity when pairs align. In every case, the program detects the blow-up and reacts by a random restart.

The third example in the rightmost column shows a null Kubo component (i.e., Δ^2^ = 0), which is a special case of boundary stalling. It is tempting to think that convergence with Δ^2^ = 0 should be possible, and examples of this are in fact observed (e.g., Video S5, trial 1, iteration 24), but there are several cases in which stalling occurs instead (e.g., Video S5, trial 5, iteration 26). Numerical analysis (not shown) reveals that stalling here is caused by inflated estimates of  stemming from near-unity covariance(s) with the null component τ_c_, which is a form of multicollinearity. This is not surprising given the uncertainty of τ_c_ is infinite for a true null component. In any case, we did not identify a scenario in which the program converged to an inaccurate fit after accounting for degenerate pairs, which implies that local minima are extremely rare for a three-level system assuming accurately phased spectra.

**Figure 5:**
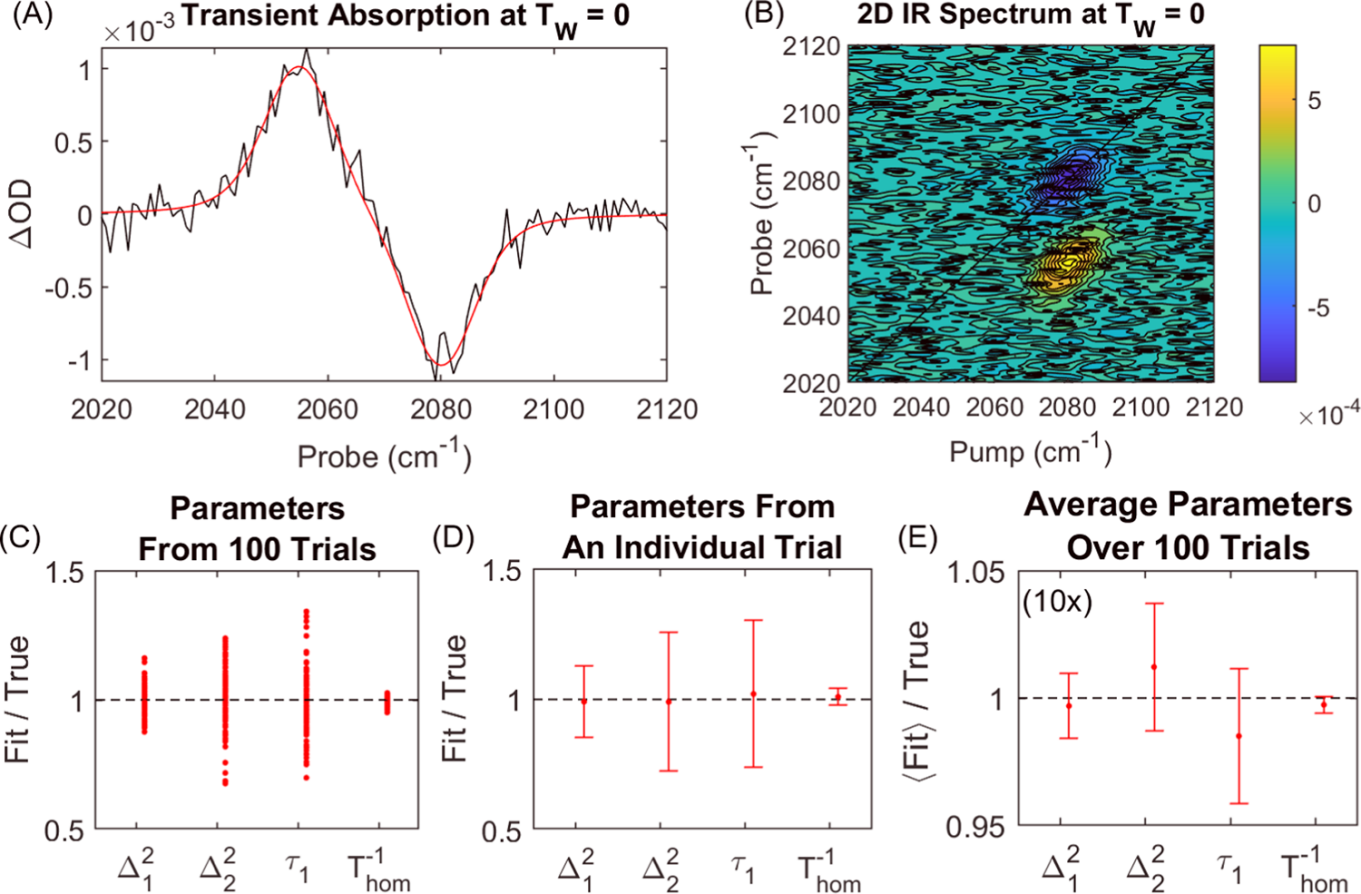
Model fitting to low SNR 2D IR waiting-time series. Simulation is representative of a cyanylated cysteine residue in the protein calmodulin, where Δ_2_^2^ is static relative to the vibrational lifetime. Examples of (A) transient absorption and (B) 2D spectrum with an SNR of 10:1. (C) Fit parameters from 100 trials. (D) Fit parameters obtained from an individual trial with 95% confidence intervals estimated from covariance of fit. (E) Average of fit parameters over all 100 trials with 95% confidence interval calculated from the standard error of the mean.

Figure 5 shows the results for fitting to data with a much lower SNR of 10:1. Simulation parameters are representative of a cyanylated cysteine residue in the calmodulin protein.^17^ The second Kubo component is treated as static on the time scale of the waiting-time measurements by holding τ_2_ constant at 1 ns. The transient absorption spectrum in panel (A) and the 2D IR spectrum in panel (B) at *T*_W_ = 0 ps illustrates just how modest the quality of the raw data is at 10:1 SNR. Dephasing parameters in panel (C) from model fitting for all 100 trials show a distribution that rarely exceeds 10–25% of the true values. Any analysis based on the CLS method would be hopeless at this 10:1 SNR. An example of dephasing parameters for an individual trial in panel (D) shows that the 95% confidence intervals accurately reflect the variance seen in panel (C), which shows that the calculated uncertainties are reliable for low SNR data. Indeed, each of the individual results for the 100 trials in panel (D) of Video S6 validates the 95% confidence intervals. Panel (E) shows the average over all 100 trials with corresponding 95% confidence intervals calculated from the standard error of the mean, confirming the accuracy of model fitting for this example. Fitting trajectories seen in Video S7 are similar to the case of MeSCN in H_2_O previously shown in Figure 1 and Video S3. Some occasions of stalling do occur, which the program readily resolves. As expected, there is a decrease in the precision of the modeling results due to the increase in the noise.

### 3.2 Fitting Experimental Data

**Figure 6:**
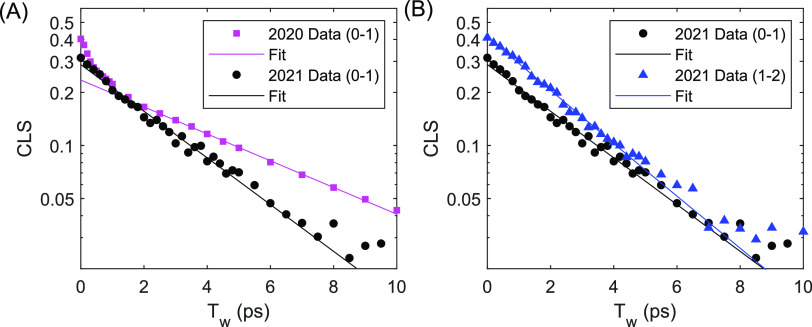
CLS of MeSCN in DMSO. (A) Comparison between 2020 data (magenta squares) and 2021 data (black circles). 2021 data are background-subtracted prior to CLS analysis, while 2020 data are not. (B) Comparison between 0–1 (black circles) and 1–2 (blue triangles) CLS for 2021 data.

For MeSCN in DMSO, we have measured and analyzed two independent data sets. One comes from our previous publication on edge-pixel referencing, and we refer to these as the 2020 data.^48^ We have also collected a new set of data that we will identify as the 2021 data. The original motivation for collecting the 2020 data was to compare data processed by two different referencing schemes. We were, therefore, not motivated to account for the background solvent response. On the other hand, in the 2021 data, the pump pulse is lower in energy by ∼8× compared to the 2020 measurements, and we have subtracted the solvent background for this measurement. The other notable differences between the data sets are the concentration (2020 is 200 mM, 2021 is 400 mM), the path length (2020 is 100 μm, 2021 is 50 μm), and that the 2020 data have significantly higher SNR due to more averaging and larger signal strength because of the higher pump energy. Figure 6A shows the results of the CLS analysis of the two data sets. Having accounted for the background response, the CLS in the 2021 data set exhibits a single exponential decay, in contrast to what is seen in the 2020 data. In addition, the CLS decay times differ by 60% between data sets (5.8 ps versus 3.3 ps) even if we only fit the long-time-scale component for the 2020 data.

Figure 6B shows the comparison of the 0–1 and 1–2 CLS decays for the 2021 data set. The two transitions have different initial CLS values (0.3 for the 0–1 and 0.4 for the 1–2), and the CLS decay of the 1–2 transition appears to be marginally faster than the 0–1 peak. We therefore model spectral diffusion of the 0–1 transition as ⟨δω_01_ (*t*)δω_01_(0)⟩ = δ(*t*)/T_hom_ + Δ^2^exp(−*t*/τ) and the 1–2 transition as ⟨δω_12_ (t)δω_12_(0)⟩ = δ(t)/T_hom_ + β^2^Δ^2^ exp(−t/τ), where β is a unitless scaling factor that accounts for the larger 1–2 CLS amplitude seen in Figure 6B. We also account for the effect that the shorter 1–2 vibrational lifetime has on the 1–2 dephasing during the second coherence time.^61,65^

**Figure 7:**
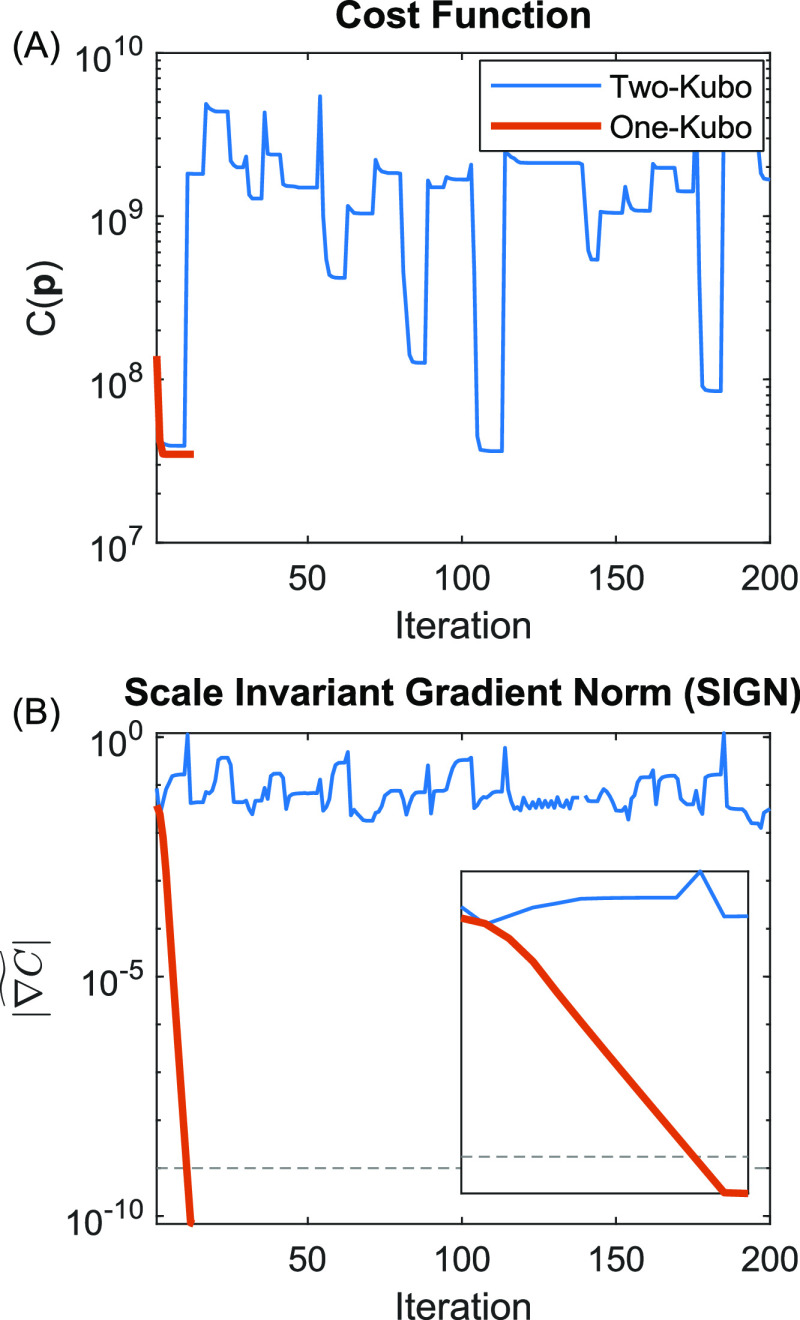
Plots of (A) cost function and (B) scale invariant gradient norm for one- (orange) and two (blue)-Kubo component models fitted to 2020 data.

Figure 7 shows plots of (A) the cost function and (B)  for model fitting to the 2020 data. A first attempt to model the data with a two-Kubo line shape (blue) does not converge and clearly resembles the results in Figure 4E,F for a model with too many Kubo components. A second attempt to model the data using a one-Kubo model (orange) immediately converges, implying that the data are best modeled by a one-Kubo line shape. Table 1 shows results for all 10 fitting variables. Our earlier analysis of the 2020 data reported a vibrational lifetime of 75 ± 4 ps, which agrees with *T*_LT_^–1^ reported by model fitting in Table 1 (inverting to 72.5 ± 0.3 ps). On the other hand, our earlier report of 5.8 ± 0.3 ps for the CLS time constant is 60% larger than the Kubo time constant reported by model fitting of the same data, 3.57 ± 0.04 ps. Importantly, however, the model fitting value of the 2020 data agrees well with the CLS time constant of 3.3 ± 0.3 ps for the 2021 data and the model fitting Kubo time constant of 3.28 ± 0.02 ps for the 2021 data.

**Table 1:** Fitting Parameters for 2020 Data

no.	parameter	fitted value
1	A_01_	16.56 ± 0.04 au
2	A_12_	18.73 ± 0.04 au
3	ω_01_	2153.319 ± 0.006 cm^–1^
4	δω_1_	0.137 ± 0.007 cm^–1^
5	Δ_Anh_	25.483 ± 0.009 cm^–1^
6	β	1.138 ± 0.003
7	*T*_LT_^–1^	0.01379 ± 0.00003 ps^–1^
8	*T*_Hom_^–1^	0.229 ± 0.003 ps^–1^
9	τ	3.57 ± 0.04 ps
10	Δ^2^	14.8 ± 0.1 cm^–2^

**Figure 8:**
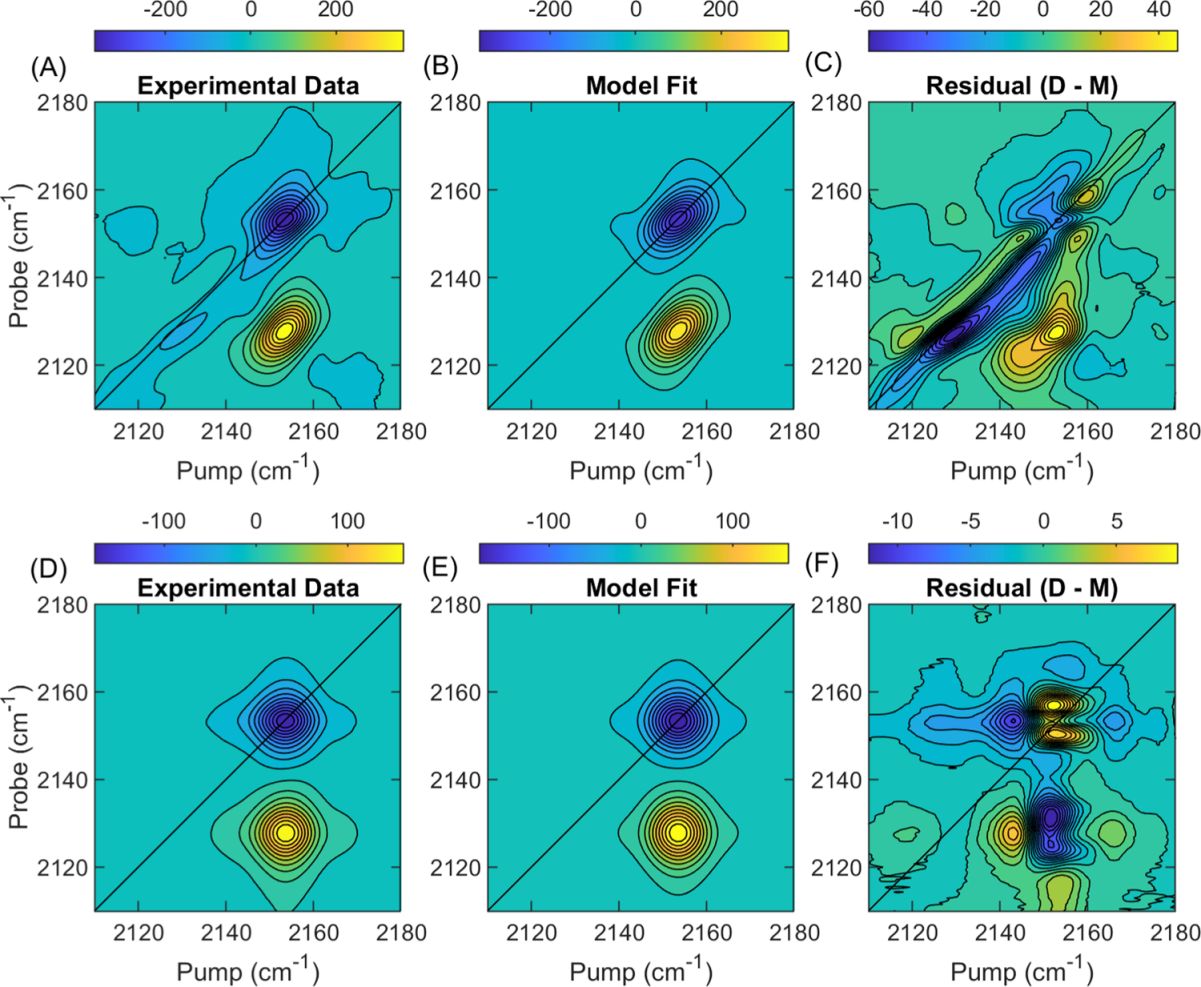
Plots of (A, D) 2020 data, (B, E) model fit result, and (C, F) residual for *T*_w_ = 0.4 ps and 50 ps. Residual in panel (C) shows a structured response, which is unaccounted for by the model.

Figure 8 shows 2D IR spectra of 2020 data in panels (A) and (D) for waiting times of 0.4 and 50 ps, and the corresponding best-fit model spectra in panels (B) and (E). Qualitatively, the model appears consistent with the data in terms of shape and scale. A closer look at the residuals in panels (C) and (F), however, reveals the presence of a structured response at both waiting times. This residual response is roughly 10% of the amplitude of the total signal and is also present at a similar magnitude in the 2021 data.

The waiting-time-dependent residuals differ in shape between the 2020 and 2021 data around the 0–1 peak (Videos S8 and S9), but for frequencies less than 2035 cm^–1^, they are similar around the diagonal, particularly at early waiting times. Differences around the 0–1 peak likely cause the discrepancies in the CLS decays shown in Figure 6A. To show this more clearly, we overlay the centerlines of the experimental spectra in the left and right panels of Videos S8 and S9. Close inspection of the right panel in Video S8 reveals the structured feature responsible for the slower CLS decay in the 2020 data, which is not present in the 2021 data (Video S9). We must caution against overinterpreting the residual here. One should not conclude that the residual reflects the “unmodeled” part of the line shape where the model **M(p)** and Jacobian **J** are nonzero. The best-fit model is influenced by both “modeled” and “unmodeled” parts of the experimental line shape such that *C*(**p**) is minimized. On the other hand, in the region below 2135 cm^–1^ along the diagonal, the residual does accurately reflect the “unmodeled” line shape because **M(p)** and **J** are virtually zero and therefore the residual **r** in this region cannot influence the model fit by eq 4.

We note three interesting features of this lower-frequency response at early waiting time. First, as shown in Videos S8 and S9, the phase of the signal oscillates as a function of waiting time with an ∼1.3 ps period (∼25 cm^–1^). Second, the signal initially appears stretched along the diagonal and dephases with a lifetime of 1–2 ps. Third, as shown in both videos, the intensity of the residual response is roughly 10% of the peak 2D IR signal in both 2020 and 2021 data, which implies a third-order response. We suspect that this may be a resonantly enhanced wave packet of low-frequency Raman modes (e.g., the methyl torsion) anharmonically coupled to the C≡N stretch, similar to what has been seen in a variety of other oscillators.^74−76^ At longer waiting times, the residual appears as a vertical peak shift of the 0–1 transition coincident with the vibrational lifetime, which is characteristic of hot ground-state absorption.^76−79^

**Figure 9:**
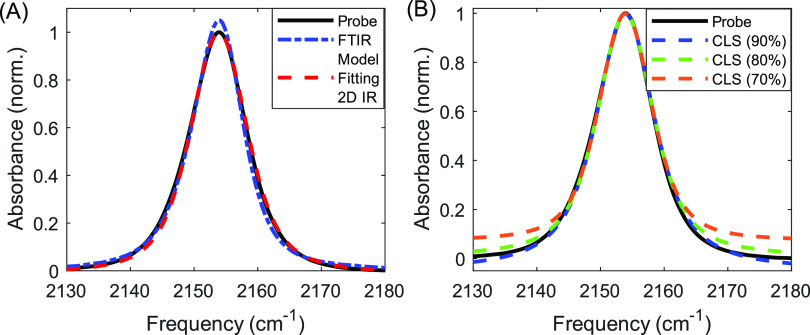
(A) Linear absorption spectra as measured by the probe (black, solid), FTIR (blue, dot-dashed), and simulated with parameters obtained by model fitting the 2D IR waiting-time series (red, dashed). (B) Results of the CLS method fitting to the upper 90% (blue, dashed), 80% (green, dashed), and 70% (orange, dashed) of the probe spectrum (black, solid) to obtain the Kubo amplitude and homogeneous dephasing.

Figure 9A shows the linear absorbance spectrum for 2021 data obtained using the probe beam and upconversion spectrometer (black, solid) and using an FTIR (blue, dashed). We subtracted DMSO backgrounds in both spectra, offset and scale the FTIR spectrum to best match the probe spectrum via linear least-squares, but the FTIR spectrum remains notably narrower than the probe spectrum. We attribute this to different instrument response functions between the two measurements. For example, the FTIR spectrum is influenced by factors such as apodization, scan length, and vignetting of light along moving optics,^70^ while the probe spectrum is influenced by the resolving power of the spectrometer and the bandwidth of the 800 nm pump utilized in upconverting the infrared light prior to the spectrometer.

The third trace in Figure 9A (red, dashed) is the linear response predicted by model fitting to the 2D IR waiting-time series (scaled and offset to best match the spectrum measured with the probe beam). The strong match between the predicted response and probe spectrum is an independent validation of model fitting. For comparison, Figure 9B shows linear absorption fits to the FTIR spectrum for the CLS method. FTIR line shapes can be unreliable near the baseline due to the difficulties with imperfect background subtraction, especially for dilute solutions of weak chromophores, and therefore, we show results for fitting to the upper 90, 80, and 70% of the linear absorbance spectrum. We float the homogeneous lifetime, Kubo amplitude, linear scaling, and offset as fitting variables while holding the Kubo correlation time constant at 3.3 ps. We see that 80% provides a reasonable balance between the quality of fit and distortions as a result of the baseline, which is in line with the recommendation by Kwak and co-workers.^14^ It is notable that the linear absorption predicted by model fitting in Figure 9A is still a higher quality fit than the 90% case for the CLS analysis.

**Figure 10:**
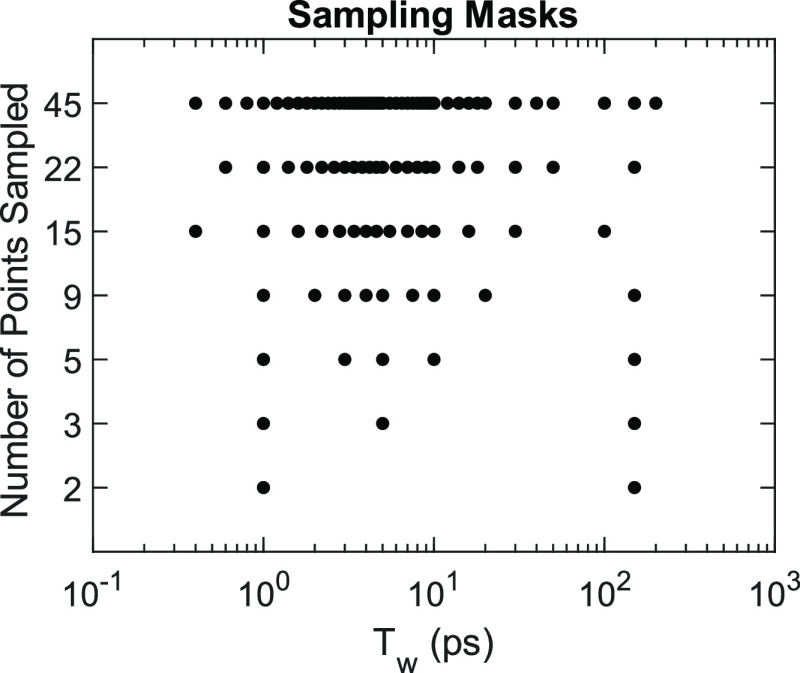
Masks used in undersampling the waiting time *T*_W_ of MeSCN in DMSO. Each row corresponds to a different sampling mask. Black dots represent the inclusion of a 2D spectrum in fitting for a given waiting time. In all cases, we exclude the first 300 fs of waiting time to avoid spurious nonresonant and time-ordering signals from pulse overlap.

We now examine the efficacy of model fitting of undersampled data with the sampling masks illustrated in Figure 10. There are 47 waiting-time spectra in the entire series, but the first two (*T*_w_ = 0 and 200 fs), where pump and probe overlap, are susceptible to spurious nonresonant and time-ordering signals. Hence, we omit these spectra from model fitting in each case and the “fully sampled” data correspond to the 45-point mask. We generate masks by keeping every *k*th point left and right of 1 ps, where *k* is an integer number. For example, the 15-point mask samples every third point left and right of 1 ps. We base this on the guiding principle that every mask should include a waiting-time point of ∼2× earlier than the shortest process expected to occur in the line shape and ∼2× later than the vibrational lifetime. Knowing a priori that the homogeneous lifetime is ∼2 ps and vibrational lifetime is ∼75 ps, we include points around 1 ps and 150 ps in every mask. For other −SCN systems more generally, one can reasonably assume that the shortest observable process is likely no faster than ∼0.8 ps and the vibrational lifetime is between 30 and 80 ps.

**Figure 11:**
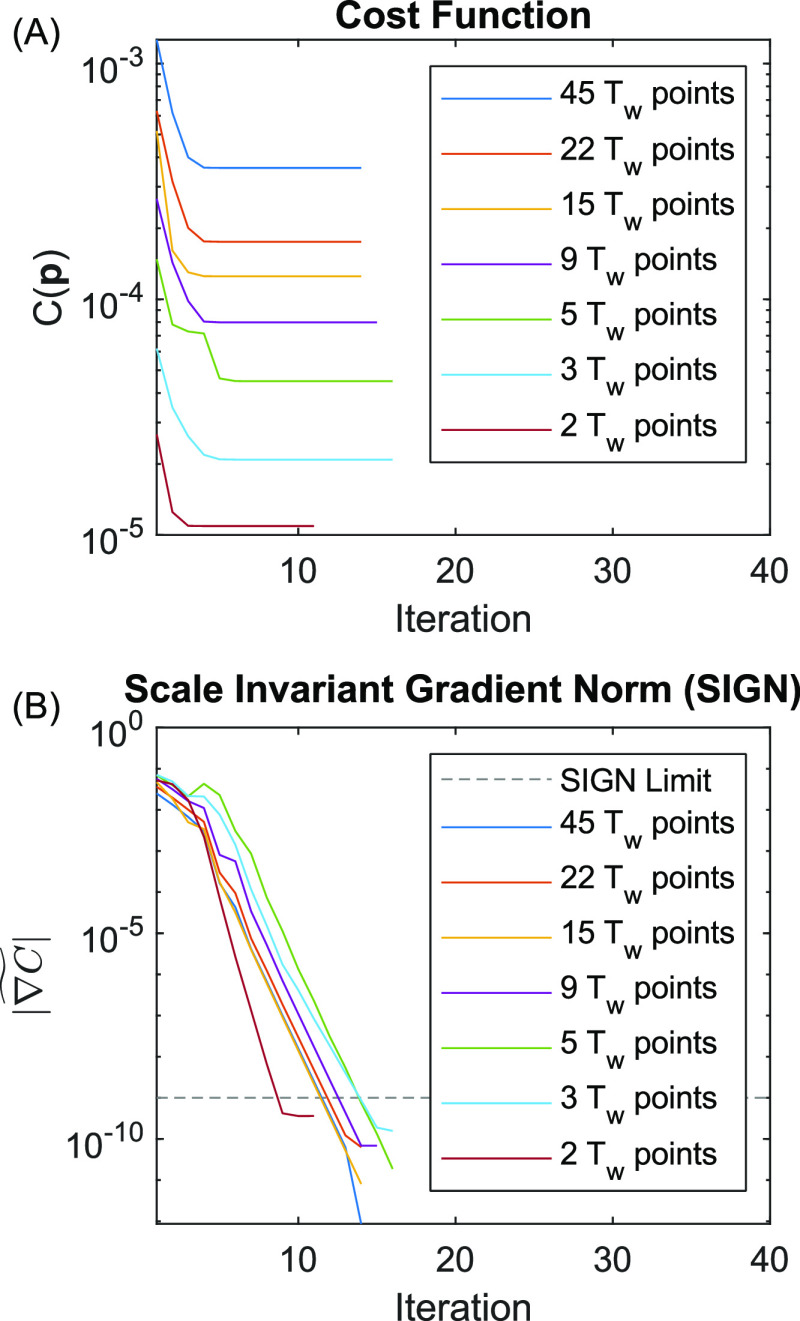
Plots of (A) the cost function C and (B) the scale invariant gradient norm  versus fitting iteration for a series of undersampled waiting time *T*_w_. The dashed line in panel (B) is associated with the stopping criterion.

Figure 11 shows plots of *C*(**p**) and  for all sampling masks. As noted earlier, the cost function scales linearly with *N*_D_, which explains the varying magnitudes of C(**p**) in Figure 11A. On the other hand, the scale invariant gradient norm has no dependence on *N*_D_, and hence,  is similar in scale for every mask.

**Figure 12:**
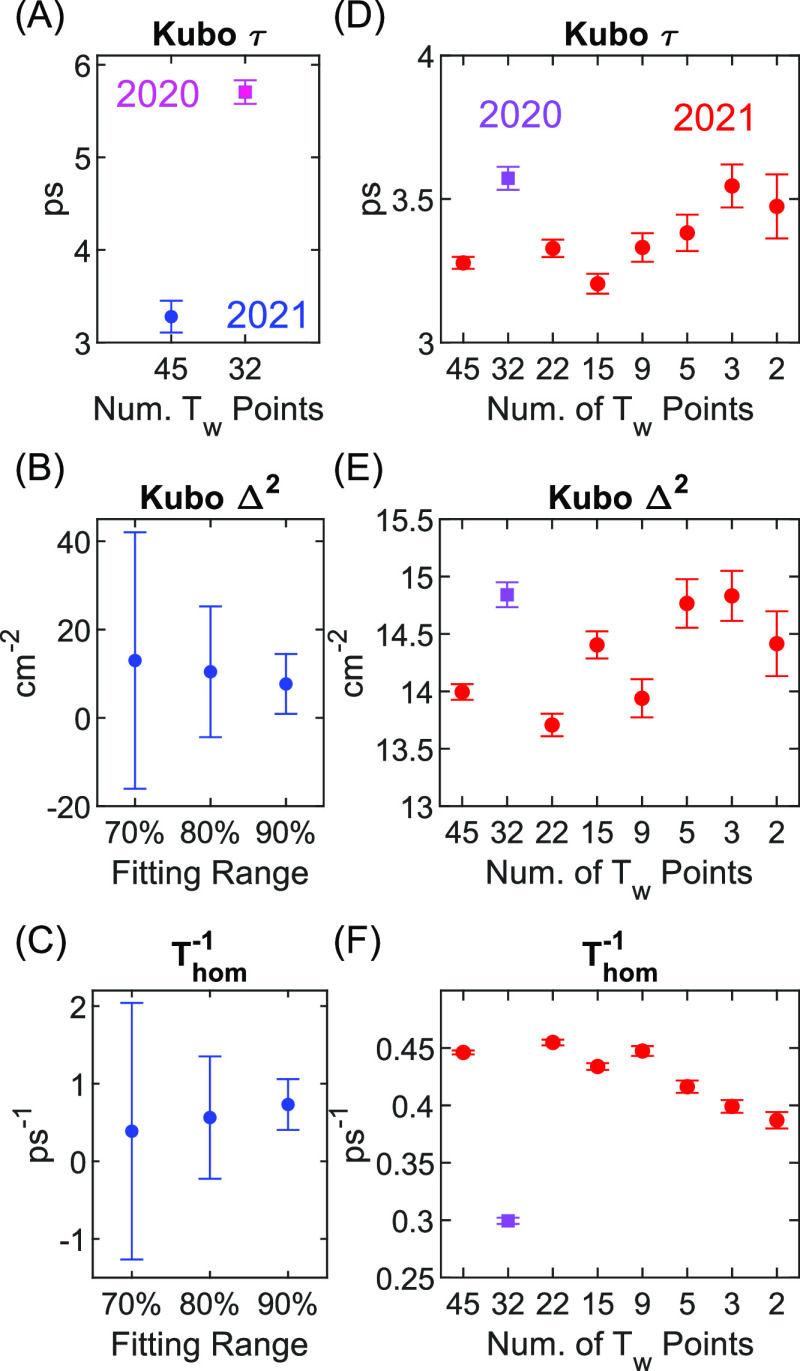
Comparison of dephasing parameters obtained by the CLS method and modeling fitting. (A) Kubo time constants obtained by the CLS method for 2020 and 2021 data. (B, C) Kubo amplitudes and homogeneous dephasing obtained by the CLS method for 2021 data. This is plotted for three different fitting ranges of linear absorption (see Figure 9B) to demonstrate how sensitive these parameters are to the linear absorption spectrum. (D–F) Model fitting of 2021 data as a function of the number of waiting-time points used in fitting. Model fitting to 2020 data also shown for comparison. Error bars are 95% confidence intervals estimated from the covariance of fit.

Figure 12 shows dephasing parameters obtained by the CLS method (left column) and model fitting for every sampling mask (right column). The 60% discrepancy between Kubo time constants in panel (A) is a reflection of the CLS decays in Figure 6A. In contrast, Kubo time constants obtained by model fitting in panel (D) differ by just 10% between the 2020 and 2021 data sets. This shows that model fitting is far more consistent and reliable for estimating Kubo time constants than the CLS method. The results in panel (D) also show that Kubo time constants are consistent across all undersampled versions of 2021 data. It is remarkable that model fitting to just two waiting-time spectra yields precision comparable to that of the CLS method for 45 waiting-time points. In practice, we do not recommend fitting to only two waiting-time spectra as we would not expect this to work for multi-Kubo line shapes.

Kubo amplitudes and homogeneous dephasing obtained by the CLS method shown in panels (B) and (C) reflect the linear absorption fits in Figure 9B. Kubo amplitudes and homogeneous dephasing obtained by model fitting in panels (E) and (F) show consistent values across all undersampled versions of 2021 data. There does appear to be a systematic depression of 15% in the homogeneous dephasing rate from left to right, but this difference is small in comparison to the uncertainty of the CLS method. Finally, results in panel (F) also show a 40% discrepancy between homogeneous dephasing rates obtained by model fitting between the 2020 and 2021 data sets. Some of this difference may be the result of background subtracting only the 2021 data set or inaccuracies in inverting the apodization window required for the 2020 data (i.e., Section 2.5). Nonetheless, this 40% discrepancy is still small in comparison to the uncertainty seen in the CLS method.

Conventional wisdom says that model fitting of multidimensional spectra is a problem riddled by local minima. In the several hundred trial examples of model fitting shown here, our program encounters and resolves many algorithmic stalls, but not a single local minimum is observed. The distinction is important. It is difficult, and often impossible, to distinguish a local minimum from the global minimum, but stalling is always distinguishable using . Though we have only shown that local minima are exceptionally rare for a simple three-level system, it is reasonable to believe that this should apply to more advanced models including coupled oscillators, assuming reasonable separation between peaks, and more complicated line-shape functions. Therefore, conventional wisdom should be updated: model fitting is far less a problem of local minima as it is of multicollinearity and boundaries, which are manageable.

### 3.3 Recommended Practices for Model Fitting

We summarize the following recommendations for model fitting.

(1) For faster performance, limit the number of data points. See Section 2.6 for suggestions.
(2) We strongly discourage superfluous zero padding or apodizing data prior to fitting as these effects will propagate into fitting results. We recommend fitting to data in the original measurement domain. See Section 2.5 for more information.
(3) When applicable, users should enable a zero-order phase fitting parameter to account for residual phasing errors (see Supporting Information Section G). The presence of spectrally correlated shot-to-shot noise (a.k.a. local-oscillator noise) likely limits the ability to accurately phase data, particularly with the projection slice theorem. Therefore, we do not recommend model fitting for phase-distorted apparatuses without prior removal of shot-to-shot noise.
(4) Calibrated referencing schemes greatly improve the precision of fitting parameters by removing correlated shot-to-shot noise. For example, we found that edge-pixel referencing reduced uncertainties by a factor of 10 over unreferenced data.
(5) The relative variance across data (e.g., due to nonuniform averaging across different waiting-time spectra) should be accounted for using **V**_D_^–1^. This is straightforward using our GUI interface. See Section 2.2 for more information.
(6) Fitting occurs simultaneously across all spectra, and therefore, consistency is required among factors that affect the magnitude of the nonlinear signal throughout the entire experiment (e.g., constant pump power, constant intensity ratio between the probe and local oscillator and consistent normalization factors if nonuniformly averaging along waiting-time spectra).
(7) For best performance, tune the initial parameters to reasonably match the model and data prior to fitting. Our GUI interface provides real-time update of the line-shape model for comparison with the data across several different plots to greatly help with this process.
(8) For best performance, boundary conditions should strike a reasonable balance between narrow enough to avoid too many random restarts and wide enough to avoid boundary stalling. Again, our GUI interface is very helpful here.
(9) Start by fitting a one-Kubo model and then add more components. For best performance with two or more components, avoid degeneracy by restricting the boundary conditions of each time component to a different time scale (e.g., 0.1–2.0 and 1.0–10 ps). Use the time constant from the one-Kubo component fit to estimate the dividing line between the two time scales with comfortable overlap to avoid overconstraining. With the exception of null components, convergence will not occur for models having more Kubo components than truly present in the data. Note that it is practically impossible to resolve a Kubo correlation time much longer than the vibrational lifetime due to low SNR at longer *T*_w_, which is similarly true for the CLS method.
10. When exploring different models, VIFs are a useful tool for monitoring multicollinearity. Plots of VIFs are readily generated in our GUI app.

## 4 Conclusions

We introduce a scale invariant gradient norm (SIGN) capable of identifying, and distinguishing between, algorithmic stalling and convergence at a local or global minimum. Our model fitting algorithm accurately estimates all line-shape parameters with superior precision and accuracy compared to the CLS method. We show how to infer when a model has too many, or too few, Kubo components for a data set based on the behavior of the SIGN. Interestingly, we find no evidence of local minima when fitting to a multi-Kubo line shape of a three-level system.

Though analysis of simulated spectra suggests that the CLS method is reliable in retrieving Kubo time constants, we have shown an experimental example in which the CLS time constants differ by 60% between independent measurements of the same system. In contrast, Kubo time constants obtained by model fitting only differ by 10%, which suggests that model fitting is a far more reliable and consistent means of measuring spectral diffusion over the CLS method. Furthermore, we revealed a fundamental oversight in the propagation of error with the CLS method, which led us to show that error bars for Kubo amplitudes and homogeneous dephasing obtained by fitting the linear absorbance spectrum are unreliable. In contrast, model fitting yields reliable error bars over a wide range of scenarios with upwards of 50× better precision than the CLS method.

While the scope of this study is limited to the isotropic response of a simple three-level system, we expect that model fitting to the anisotropic response should work equally as well. We plan to explore this in a follow-up study in addition to more complicated line-shape models, such as coupled oscillators, overlapping ensembles, and underdamped oscillatory FFCFs.

## Supporting Information Available

URLs to videos referenced; example of Kubo line-shape asymmetry; details of line-shape model; derivation of the variance–covariance matrix for model fitting; derivation of the modified variance–covariance matrix for the CLS method; comparison between referenced and unreferenced data; model fitting with phasing errors; and instructions for reproducing results and figures (PDF)
