# Supplementary material for: Least-Squares Fitting of Multidimensional Spectra to Kubo Line-Shape Models
Source: J Phys Chem B. 2021 Nov 16;125(46):12876–91. doi: 10.1021/acs.jpcb.1c08764 (PMC8630800; doi:10.1021/acs.jpcb.1c08764)
Supplement: Supplementary file 1 — jp1c08764_si_001.pdf [file jp1c08764_si_001.pdf]

# Supporting Information

## Least-Squares Fitting of Multidimensional Spectra to Kubo Lineshape Models

Kevin C. Robben[a], Christopher M. Cheatum[a]*

[a]*Department of Chemistry, University of Iowa, Iowa City, Iowa 52242, USA*

*Author to whom correspondence should be sent. E-mail: christopher-cheatum@uiowa.edu

Contents

A. Videos ... 2
B. Asymmetry of Kubo Lineshape ... 4
C. Lineshape Model ... 4
D. Derivation of the Variance-Covariance Matrix ... 6
E. The Modified Variance-Covariance Matrix for the CLS Method ... 7
F. Comparison Between Referenced and Unreferenced Data ... 8
G. Model Fitting with Phasing Errors ... 9
H. Instructions for Reproducing Results and Figures ... 10
I. References ... 12

## A. Videos

Videos referenced in the main text are available at the following unlisted YouTube links.

**Video S1**: https://youtu.be/7FJCR0fogkk
Description: **Biexponential fits to the CLS decay** for 100 trials of simulated 2D IR waiting-time spectra of MeSCN in $H_2O$ (**SNR 600:1**) based on lineshape parameters reported by Yuan and Fayer.[1]

**Video S2**: https://youtu.be/3eqEChFN_E4
Description: Model fitting results plotted as **fit/true** ratio for 100 trials of simulated 2D IR waiting-time spectra of MeSCN in $H_2O$ (**SNR 600:1**) based on lineshape parameters reported by Yuan and Fayer.[1]

**Video S3**: https://youtu.be/OcwDMr8RQjE
Description: Recording of model fitting update window, which includes **fitting trajectories** using 10/12 parameters for 100 trials of simulated 2D IR waiting-time spectra of MeSCN in $H_2O$ (**SNR 600:1**) based on lineshape parameters reported by Yuan and Fayer.[1]

**Video S4**: https://youtu.be/UL23pn9Py78
Description: Recording of model fitting update window, which includes fitting trajectories using 8/10 parameters for 100 trials of 2D IR waiting-time spectra simulated by a **two-Kubo lineshape** but fit to a **one-Kubo model** (too few components to fit the data).

**Video S5**: https://youtu.be/nd1Ttuke0Hw
Description: Recording of model fitting update window, which includes fitting trajectories using 10/12 parameters 100 trials of 2D IR waiting-time spectra simulated by a **one-Kubo lineshape** but fit to a **two-Kubo model** (too many components to fit the data).

**Video S6**: https://youtu.be/wcDJQKpECO0
Description: Model fitting results plotted as **fit/true ratio** for 100 trials of simulated 2D IR waiting-time spectra representative of a cyanylated cysteine reporter in Calmodulin protein (**SNR 10:1**) based on lineshape parameters reported by Schmidt-Engler and coworkers.[2]

**Video S7**: https://youtu.be/9Hz_EU6m53k
Description: Recording of model fitting update window, which includes **fitting trajectories** using 10/12 parameters, for 100 trials of 2D IR waiting-time spectra representative of a cyanylated cysteine reporter in Calmodulin protein (**SNR 10:1**) based on parameters reported by Schmidt-Engler and coworkers.[2]

**Video S8**: https://youtu.be/1BPlzRgPiGE
Description: Comparative plots of experimental (left), model fit (middle), and residual (right) 2D IR waiting-time spectra of MeSCN in DMSO collected in **2020**.[3] Yellow dots

on left panel indicate the center-line of the 2020 experimental data. We overlay the same center-lines onto the residual spectra on the right panel as an aide in understanding how the experimental lineshape differs from the best fit model and how this residual difference might explain why the 2020 CLS decay is so different from the 2021 CLS decay.

**Video S9**: https://youtu.be/WxwiD7DwLTw
Description: Comparative plots of experimental (left), model fit (middle), and residual (right) 2D IR waiting-time spectra of MeSCN in DMSO collected in **2021**. Yellow dots on left panel indicate the center-line of the 2020 experimental data. We overlay the same center-lines onto the residual spectra on the right panel as an aide in understanding how the experimental lineshape differs from the best fit model and how this residual difference might explain why the 2020 CLS decay is so different from the 2021 CLS decay.

**Video S10**: https://youtu.be/kIbpuhalFzY
Description: Recording of model fitting update window, which includes **fitting trajectories** of 10/12 parameters, for 100 trials of 2D IR waiting-time spectra representative of a cyanylated cysteine reporter in Calmodulin protein (SNR 100:1) based on parameters reported by Schmidt-Engler and coworkers,[2] **including phasing errors** (RMSD 6°) but **without** a phasing error fitting parameter.

**Video S11**: https://youtu.be/MdaV4_uKzZU
Description: Model fitting results plotted as **fit/true** ratio for 100 trials of simulated 2D IR waiting-time spectra representative of a cyanylated cysteine reporter in Calmodulin protein (SNR 100:1) based on parameters reported by Schmidt-Engler and coworkers,[2] **including phasing errors** (RMSD 6°) but **without** a phasing error fitting parameter.

**Video S12**: https://youtu.be/pvm2vhSMm7k
Description: Recording of model fitting update window, which includes **fitting trajectories** of 10/13 parameters, for 100 trials of 2D IR waiting-time spectra representative of a cyanylated cysteine reporter in Calmodulin protein (SNR 100:1) based on parameters reported by Schmidt-Engler and coworkers,[2] **including phasing errors** (RMSD 6°) and **fitting parameter ϕ** to account for phasing error.

**Video S13**: https://youtu.be/3XQO1OYDWHE

Description: Model fitting results plotted as **fit/true** ratio for 100 trials of simulated 2D IR waiting-time spectra representative of a cyanylated cysteine reporter in Calmodulin protein (SNR 100:1) based on parameters reported by Schmidt-Engler and coworkers,[2] **including phasing errors** (RMSD 6°) and **fitting parameter ϕ** to account for phasing error.

## B. Asymmetry of Kubo Lineshape

As shown in Figure S1., the generic 2D Kubo lineshape has a frequency dependent asymmetry to it. As a result, fitting a symmetric function (e.g. Lorentzian, Gaussian, Voigt) will yield inaccurate center-lines for CLS method. We therefore prefer asymmetric fitting functions for measuring the center-line (e.g. a Lorentzian + linear term).

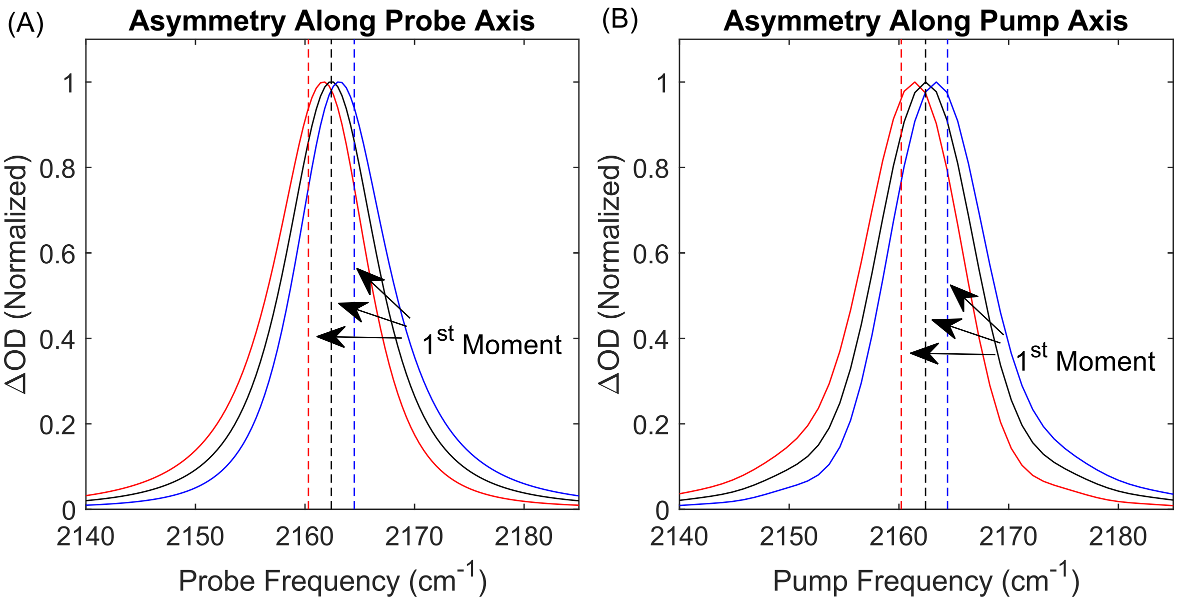


Figure S1. Asymmetry of generic 2D Kubo lineshape for a two-level system (omitting the anharmonic peak). (A) Slices of 0-1 peak along the probe axis for a 2D Kubo lineshape at early waiting time taken at three different pump frequencies (red is left of center, black is center, blue is right of center). The first moment of each slice is illustrated by a vertical band. Results clearly show frequency dependent asymmetry of the Kubo lineshape. (B) Same as for (A) but now along the pump axis.

## C. Lineshape Model

The usual treatment for computing the 2D IR spectrum in the frequency-frequency domain is to first compute the rephasing spectrum in the $(t_1, t_3) = (-, +)$ quadrant and the nonrephasing spectrum in the $(t_1, t_3) = (+, +)$ quadrant, then FFT along the pump axis $(t_1 \rightarrow \omega_1)$, and finally FFT along the probe axis $(t_3 \rightarrow \omega_3)$. For model fitting in the $(t_1, \omega_3)$ domain, this would require a total of three FFT's. We prefer the more direct route shown in Eq. S1 and Eq. S2 where we compute the rephasing spectrum in the $(t_1, t_3) = (+, -)$ quadrant. This ensures $t_1 \geq 0$ and requires only one FFT along the probe axis $(t_3 \rightarrow \omega_3)$ to reach the $(t_1, \omega_3)$ domain. Here $R_+$ denotes nonrephasing, $R_-$ denotes rephasing and $\phi$ accounts for a symmetric phasing error when applicable. Note that $R_+$ and $R_-$ are analytic response functions, and therefore DC components require multiplication by ½ prior to FFT.[4]

$$\mathrm{FID}(t_1, T_W, \omega_3) = \int_{-\infty}^{\infty} R(t_1, T_W, t_3) e^{-i2\pi c \omega_3 t_3} dt_3 \qquad \text{Eq. S1}$$

$$R(t_1, T_W, t_3) = \begin{cases} e^{+i\phi} R_+(t_1, T_W, t_3) & \text{for } t_3 > 0 \\ e^{-i\phi} R_-(t_1, T_W, t_3) & \text{for } t_3 < 0 \\ \left(e^{+i\phi} R_+(t_1, T_W, t_3) + e^{-i\phi} R_-(t_1, T_W, t_3)\right)/2 & \text{for } t_3 = 0 \end{cases} \qquad \text{Eq. S2}$$

For simplicity, we assume stimulated emission and ground state bleach are equivalent, and hence, $R_\pm$ is given by Eq. S3 where ESA denotes excited state absorption and GSB denotes ground state bleach.

$$R_\pm(t_1, T_W, t_3) = ESA_\pm(t_1, T_W, t_3) - GSB_\pm(t_1, T_W, t_3) \qquad \text{Eq. S3}$$

Because computers use floating-point arithmetic to do math, we expect small numerical errors. In general, the following guidelines are recommended to minimize floating-point errors:[5]

1. We prefer Multiplication and Division, which are the most reliable operations.
2. We avoid Addition and Subtraction whenever possible, which are particularly susceptible to roundoff errors when:
   a. The two values differ by many orders of magnitude.
   b. The two values are nearly equal and subtracted (actually, this one is so egregious it has its own name: cancellation error).
3. We limit the number of operations as much as possible because floating-point errors propagate and magnify with every operation.

In fact, given enough calculations, floating-point error can grow so large that a fitting problem may become ill-conditioned just from the accumulated error alone. Though we have not confirmed that this is critical in computing nonlinear lineshape functions, we note that the common written expression for the multidimensional Kubo lineshape is ideal for floating-point errors (lots of addition and subtraction!). Therefore, in programming lineshape functions, we exercise caution and consider the three guidelines above. Their expression below accurately reflects our MATLAB code, though we acknowledge that our expressions may not be the "best" or most "stable" means of computing the lineshapes.

The full lineshape for GSB is shown in Eq. S4 where $\omega_{01}$ is the 0-1 center frequency, $\omega_{s3}$ is the DC-shift frequency along the probe axis, $\omega_{s1}$ is the DC-shift frequency along the pump axis, and $\delta\omega_1$ is the calibration mismatch error between the pump and probe axis (typically $< 1\ \text{cm}^{-1}$). The lineshape for ESA is provided by Eq. S5 where $\Delta_{Anh}$ is the anharmonicity.

$$\begin{aligned} GSB_\pm(t_1, T_W, t_3) &= A_{01} e^{+i2\pi c t_3(\omega_{01} - \omega_{s3}) \pm i2\pi c t_1(\omega_{01} - \omega_{s1} - \delta\omega_1)} \\ &\times G_{Hom(01)}(t_1, T_W, t_3) \times \prod_{i=1}^{N_{Kubo}} G_{Kubo(i)}(t_1, T_W, t_3, \pm, 1) \end{aligned} \qquad \text{Eq. S4}$$

$$\begin{aligned} ESA(t_1, T_W, t_3, \pm) &= A_{12} e^{+i2\pi c t_3(\omega_{01} - \omega_{s3} - \Delta_{Anh}) \pm i2\pi c t_1(\omega_{01} - \omega_{s1} - \delta\omega_1)} \\ &\times G_{Hom(12)}(t_1, T_W, t_3) \times \prod_{i=1}^{N_{Kubo}} G_{Kubo(i)}(t_1, T_W, t_3, \pm, \beta) \end{aligned} \qquad \text{Eq. S5}$$

Notice that homogeneous contributions, $G_{Hom(01)}$ in Eq. S6 and $G_{Hom(12)}$ in Eq. S7, differ due to 1-2 lifetime broadening during $t_3$ coherence.[6, 7]

$$G_{Hom(01)}(t_1, T_W, t_3) = \exp(-T_{Hom}^{-1}t_1)\exp(-T_{LT}^{-1}T_W)\exp(-T_{Hom}^{-1}t_3) \qquad \text{Eq. S6}$$

$$\begin{aligned} &G_{Hom(12)}(t_1, T_W, t_3) \\ &\quad = \exp(-T_{Hom}^{-1}t_1)\exp(-T_{LT}^{-1}T_W)\exp(-(T_{Hom}^{-1} + T_{LT}^{-1})t_3) \end{aligned} \qquad \text{Eq. S7}$$

The Kubo contribution is given by Eq. S8, in which $\beta$ the anharmonic scaling factor for the 1-2 FFCF relative to the 0-1 FFCF (for GB, $\beta = 1$).

$$\begin{aligned} &G_{Kubo(i)}(t_1, T_W, t_3, \pm, \beta) \\ &\quad = \exp\left(-(2\pi c)^2\Delta_i^2\tau_i^2\left(\mathrm{expm1}\left(\frac{t_1}{\tau_i}\right) + \frac{t_1}{\tau_i}\right)\right) \\ &\quad \times \exp\left(-(2\pi c)^2\beta^2\Delta_i^2\tau_i^2\left(\mathrm{expm1}\left(\frac{t_3}{\tau_i}\right) + \frac{t_3}{\tau_i}\right)\right) \\ &\quad \times \exp\left(\mp\beta\Delta_i^2\tau_i^2\exp\left(\frac{T_W}{\tau_i}\right)\mathrm{expm1}\left(\frac{t_1}{\tau_i}\right)\mathrm{expm1}\left(\frac{t_3}{\tau_i}\right)\right) \end{aligned} \qquad \text{Eq. S8}$$

As noted above, these expressions differ from the usual presentation in literature in order to accurately reflect our programming, which considers the guidelines above for minimizing floating-point error. Note that expm1 is a standard function dedicated to computing a stable form of "$\exp(x) - 1$", which is otherwise susceptible to cancellation error near $x = 0$.

## D. Derivation of the Variance-Covariance Matrix

We denote true parameters as $\mathbf{p}^*$ $(N_p \times 1)$ and a perturbation in data (e.g. noise) as $\mathbf{dD}$ $(N_D \times 1)$ such that $\mathbf{D} = \mathbf{M}(\mathbf{p}^*) + \mathbf{dD}$. For an unperturbed data set ($\mathbf{dD} = \mathbf{0}$) located at the global minimum ($\mathbf{p} = \mathbf{p}^*$), we have that $\boldsymbol{\nabla}_\mathbf{p}\mathbf{C} = \mathbf{0}$ by definition of a minimum (in the main text, $\boldsymbol{\nabla}\mathbf{C}$ is equivalent to $\boldsymbol{\nabla}_\mathbf{p}\mathbf{C}$). Now suppose the data is perturbed ($\mathbf{dD} \neq \mathbf{0}$) which shifts the global minimum to $\mathbf{p}^* + \mathbf{dp}$. Then for infinitesimally small $\mathbf{dD}$, the total differential of $\boldsymbol{\nabla}_\mathbf{p}\mathbf{C}$ in Eq. S9 relates $\mathbf{dp}$ and $\mathbf{dD}$ via $\mathrm{d}\left(\boldsymbol{\nabla}_\mathbf{p}\mathbf{C}(\mathbf{p}, \mathbf{D})\right) = 0$, which is true because $\boldsymbol{\nabla}_\mathbf{p}\mathbf{C}(\mathbf{p}, \mathbf{D}) = 0$ at both minima.

$$\mathrm{d}\left(\boldsymbol{\nabla}_\mathbf{p}\mathbf{C}(\mathbf{p}, \mathbf{D})\right) = \boldsymbol{\nabla}_\mathbf{p}\left(\boldsymbol{\nabla}_\mathbf{p}\mathbf{C}\right)\mathbf{dp} + \boldsymbol{\nabla}_\mathbf{D}\left(\boldsymbol{\nabla}_\mathbf{p}\mathbf{C}\right)\mathbf{dD} = \mathbf{0} \qquad \text{Eq. S9}$$

We rearrange Eq. S9 to show that $\mathbf{dp} = -\left(\boldsymbol{\nabla}_\mathbf{p}^2\mathbf{C}\right)^{-1}\left(\boldsymbol{\nabla}_\mathbf{D}\boldsymbol{\nabla}_\mathbf{p}\mathbf{C}\right)\mathbf{dD}$ assuming $\boldsymbol{\nabla}_\mathbf{p}^2\mathbf{C}$ is invertible. To compute $\boldsymbol{\nabla}_\mathbf{D}\boldsymbol{\nabla}_\mathbf{p}\mathbf{C}$, we must apply $\boldsymbol{\nabla}_\mathbf{D}$ to $\boldsymbol{\nabla}_\mathbf{p}\mathbf{C} = -2\mathbf{r}^T\mathbf{V}_\mathbf{D}^{-1}\mathbf{J}$. The residual $\mathbf{r}^T = (\mathbf{D} - \mathbf{M})^\mathbf{T}$ is the only factor in $\boldsymbol{\nabla}_\mathbf{p}\mathbf{C}$ that depends on $\mathbf{D}$. Given that $\boldsymbol{\nabla}_\mathbf{D}(\mathbf{r}^\mathbf{T})$ is equal to the identity matrix, it follows that $\boldsymbol{\nabla}_\mathbf{D}\boldsymbol{\nabla}_\mathbf{p}\mathbf{C} = \boldsymbol{\nabla}_\mathbf{D}(-2\mathbf{r}^T\mathbf{V}_\mathbf{D}^{-1}\mathbf{J}) = -2\mathbf{J}^T\mathbf{V}_\mathbf{D}^{-1}$ where an extra transpose is necessary when evaluating $\boldsymbol{\nabla}_\mathbf{D}$ to ensure the dimensions of $\boldsymbol{\nabla}_\mathbf{D}\boldsymbol{\nabla}_\mathbf{p}\mathbf{C}$ $(N_p \times N_D)$ are compatible with multiplication by $\mathbf{dD}$ $(N_D \times 1)$. The differential occurs about the unperturbed coordinates where $\mathbf{r} = \mathbf{0}$, so we evaluate $\boldsymbol{\nabla}_\mathbf{p}^2\mathbf{C} = 2\left(\mathbf{J}^T\mathbf{V}_\mathbf{D}^{-1}\mathbf{J} - \mathbf{H}\right) = 2\mathbf{J}^T\mathbf{V}_\mathbf{D}^{-1}\mathbf{J}$ where $\mathbf{H}_{j,k} = \mathbf{r}^T\mathbf{V}_\mathbf{D}^{-1}\partial^2\mathbf{M}/\partial p_j\partial p_k = 0$. Upon substitution we find $\mathbf{dp}$ is linearly related to the perturbation $\mathbf{dD}$ by Eq. S10 (i.e. error propagates *linearly* from the data to fit parameters).

$$\mathbf{dp} = \left(\mathbf{J}^{\mathrm{T}}\mathbf{V}_{\mathbf{D}}^{-1}\mathbf{J}\right)^{-1}\mathbf{J}^{\mathrm{T}}\mathbf{V}_{\mathbf{D}}^{-1}\mathbf{dD} \qquad \text{Eq. S10}$$

Eq. S10 is easy to test. One may conjure up a model $\mathbf{M}$, add to it any arbitrary noise or perturbation $\mathbf{dD}$ (with $\mathbf{V}_{\mathbf{D}}^{-1} \propto \langle \mathbf{dDdD}^{\mathbf{T}} \rangle^{-1}$), and confirm by model fitting that the perturbation in the fit $\mathbf{p}$ is correctly predicted by Eq. S10 for sufficiently small noise. See section H below for instruction for verifying Eq. S10 using the model fitting GUI.

Computing $\mathbf{V}_{\mathbf{p}}$ is straightforward now: $\mathbf{V}_{\mathbf{p}} \stackrel{\text{def}}{=} \langle \mathbf{dpdp}^{\mathbf{T}} \rangle$. That is, the *parameter* variance-covariance matrix $\mathbf{V}_{\mathbf{p}}$ is the expectation of the outer product $\mathbf{dpdp}^{\mathbf{T}}$ where we denote the expectation of noise by brackets. The outer product $\langle \mathbf{dDdD}^{\mathbf{T}} \rangle$ (of size $\mathrm{N_D} \times \mathrm{N_D}$) is known as the *data* variance covariance matrix ($\propto \mathbf{V}_{\mathbf{D}}$). Note that for a data set of size $256 \times 64 \times 64$, the triangle form of $\langle \mathbf{dDdD}^{\mathbf{T}} \rangle$ occupies 1 terabyte of memory. It's well known that noise is in fact covariant for multidimensional spectroscopies utilizing pulsed lasers (particularly along the probe axis), but accounting for this is clearly impractical. However, it is worth noting that $\langle \mathbf{dDdD}^{\mathbf{T}} \rangle$ could be well approximated by a sparse matrix which only accounts for the covariance along the probe axis per individual waiting time spectrum, which could reduce the memory footprint of $\langle \mathbf{dDdD}^{\mathbf{T}} \rangle$ well enough to make this a tractable problem.

Now let $\mathbf{V}_{\mathbf{D}}^{-1} = \alpha \langle \mathbf{dDdD}^{\mathbf{T}} \rangle^{-1}$ where α is an arbitrary proportionality constant. Then $\mathbf{V}_{\mathbf{p}} \stackrel{\text{def}}{=} \langle \mathbf{dpdp}^{\mathbf{T}} \rangle = \left(\mathbf{J}^{\mathrm{T}}\mathbf{V}_{\mathbf{D}}^{-1}\mathbf{J}\right)^{-1}\mathbf{J}^{\mathbf{T}}\left(\mathbf{V}_{\mathbf{D}}^{-1}\right)\langle \mathbf{dDdD}^{\mathbf{T}} \rangle\left(\mathbf{V}_{\mathbf{D}}^{-1}\right)^{\mathbf{T}}\mathbf{J}\left(\mathbf{J}^{\mathrm{T}}\mathbf{V}_{\mathbf{D}}^{-1}\mathbf{J}\right)^{-1} = \alpha\left(\mathbf{J}^{\mathrm{T}}\mathbf{V}_{\mathbf{D}}^{-1}\mathbf{J}\right)^{-1}$, and $\mathrm{C} = \mathbf{r}^{\mathbf{T}}\mathbf{V}_{\mathbf{D}}^{-1}\mathbf{r} = \alpha\mathbf{r}^{\mathbf{T}}\langle \mathbf{dDdD}^{\mathbf{T}} \rangle^{-1}\mathbf{r} \approx \alpha(\mathrm{N_D} - \mathrm{N_p})$.[a] Now substituting the expression $\alpha \approx \mathrm{C}/(\mathrm{N_D} - \mathrm{N_p})$ into $\langle \mathbf{dpdp}^{\mathbf{T}} \rangle = \alpha\left(\mathbf{J}^{\mathrm{T}}\mathbf{V}_{\mathbf{D}}^{-1}\mathbf{J}\right)^{-1}$, we find the formula for $\mathbf{V}_{\mathbf{p}}$ in Eq. S11.

$$\mathbf{V}_{\mathbf{p}} = \frac{\mathrm{C}}{\mathrm{N_D} - \mathrm{N_p}}\left(\mathbf{J}^{\mathrm{T}}\mathbf{V}_{\mathbf{D}}^{-1}\mathbf{J}\right)^{-1} \qquad \text{Eq. S11}$$

## E. The Modified Variance-Covariance Matrix for the CLS Method

The derivation of the *modified* covariance matrix is a simple extension of the covariance derivation above. As before, let true parameters be denoted as $\mathbf{p}^*$. As an example, consider the problem of fitting the linear absorption spectrum via the CLS method with a two-Kubo component model plus a homogeneous dephasing component. Furthermore, in keeping with the MATLAB example located at Manuscript Examples\CLS Propagation of Error\TestSIEq13.m, we define the order of the fitting variables as $\mathbf{p}^* = [\mathrm{A_{01}}; \mathrm{c}; \Delta_1^2; \Delta_2^2; \mathrm{T_{Hom}^{-1}}]$ implying $\mathrm{N}_{\mathbf{p}} = 5$. Then the shift $\mathbf{dp}$ of the global minimum away from $\mathbf{p}^*$ due to the addition of noise $\mathbf{dD}$ in the linear absorbance spectrum and error $\mathbf{d\tau}$ in measuring the Kubo time constants by fitting the CLS decay, are related by the following total differential.

$$\mathrm{d}\left(\boldsymbol{\nabla}_{\mathbf{p}}\mathrm{C}(\mathbf{p}, \mathbf{D}, \boldsymbol{\tau})\right) = \boldsymbol{\nabla}_{\mathbf{p}}\left(\boldsymbol{\nabla}_{\mathbf{p}}\mathrm{C}\right)\mathbf{dp} + \boldsymbol{\nabla}_{\mathbf{D}}\left(\boldsymbol{\nabla}_{\mathbf{p}}\mathrm{C}\right)\mathbf{dD} + \boldsymbol{\nabla}_{\boldsymbol{\tau}}\left(\boldsymbol{\nabla}_{\mathbf{p}}\mathrm{C}\right)\mathbf{d\tau} = \mathbf{0} \qquad \text{Eq. S12}$$

---

[a] Here $\mathbf{dD}$ corresponds to the true noise while $\mathbf{r}$ corresponds to the residual which approximately resembles the true noise at the global minimum. However, the *residual* variance at the global minimum is smaller than the *true* variance by roughly $\mathrm{N_p}$ degrees of freedom, and hence the minus $\mathrm{N_p}$ term.

This is rearranged to find $\mathbf{dp} = -\left(\boldsymbol{\nabla}_{\mathbf{p}}^{2}\mathbf{C}\right)^{-1}\left(\boldsymbol{\nabla}_{\mathbf{D}}\boldsymbol{\nabla}_{\mathbf{p}}\mathbf{C}\mathbf{dD} + \boldsymbol{\nabla}_{\boldsymbol{\tau}}\boldsymbol{\nabla}_{\mathbf{p}}\mathbf{C}\mathbf{d}\boldsymbol{\tau}\right)$. Gradients are evaluated at the unperturbed coordinates $\mathbf{p} = \mathbf{p}^{*}$, $\mathbf{D} = \mathbf{M}(\mathbf{p}^{*})$ (i.e. $\mathbf{r} = \mathbf{0}$), and $\boldsymbol{\tau} = \boldsymbol{\tau}^{*}$, and therefore $\boldsymbol{\nabla}_{\mathbf{p}}^{2}\mathbf{C} = 2\mathbf{J}_{\mathbf{p}}^{\mathbf{T}}\mathbf{J}_{\mathbf{p}}$, $\boldsymbol{\nabla}_{\mathbf{D}}\boldsymbol{\nabla}_{\mathbf{p}}\mathbf{C} = -2\mathbf{J}_{\mathbf{p}}^{\mathbf{T}}$ and $\boldsymbol{\nabla}_{\boldsymbol{\tau}}\boldsymbol{\nabla}_{\mathbf{p}}\mathbf{C} = 2\mathbf{J}_{\mathbf{p}}^{\mathbf{T}}\mathbf{J}_{\boldsymbol{\tau}}$ where $\mathbf{J}_{\mathbf{p}}$ ($\mathrm{N_D} \times \mathrm{N_p}$) and $\mathbf{J}_{\boldsymbol{\tau}}$ ($\mathrm{N_D} \times 2$) are the Jacobians of the linear absorbance spectrum with respect to $\mathbf{p}$ and $\boldsymbol{\tau}$, respectively, and we have assumed $\mathbf{V}_{\mathbf{D}}^{-1} = \mathbb{1}$ for simplicity. We rewrite $\mathbf{dp}$ in terms of the augmented matrix $\left[\mathbf{J}_{\mathbf{p}}^{\mathbf{T}} \mid -\mathbf{J}_{\mathbf{p}}^{\mathbf{T}}\mathbf{J}_{\boldsymbol{\tau}}\right]$ $\left(\mathrm{N}_{\mathbf{p}} \times (\mathrm{N_D} + 2)\right)$ and augmented vector $\left[\frac{\mathbf{dD}}{\mathbf{d}\boldsymbol{\tau}}\right]$ $\left((\mathrm{N_D} + 2) \times 1\right)$ as follows:

$$\mathbf{dp} = \left(\mathbf{J}_{\mathbf{p}}^{\mathbf{T}}\mathbf{J}_{\mathbf{p}}\right)^{-1}\left[\mathbf{J}_{\mathbf{p}}^{\mathbf{T}} \mid -\mathbf{J}_{\mathbf{p}}^{\mathbf{T}}\mathbf{J}_{\boldsymbol{\tau}}\right]\left[\frac{\mathbf{dD}}{\mathbf{d}\boldsymbol{\tau}}\right] \qquad \text{Eq. S13}$$

See section H below for instructions to verify the accuracy of Eq. S13. Having derived $\mathbf{dp}$ in Eq. S13, the covariance matrix, $\mathbf{cov}(\mathbf{p}) \stackrel{\text{def}}{=} \langle\mathbf{dpdp}^{\mathbf{T}}\rangle$, is simply given by:

$$\mathbf{cov}(\mathbf{p}) = \left(\mathbf{J}_{\mathbf{p}}^{\mathbf{T}}\mathbf{J}_{\mathbf{p}}\right)^{-1}\left[\mathbf{J}_{\mathbf{p}}^{\mathbf{T}} \mid -\mathbf{J}_{\mathbf{p}}^{\mathbf{T}}\mathbf{J}_{\boldsymbol{\tau}}\right]\mathbf{diag}(\mathbf{dD}^{2}|\mathbf{d}\boldsymbol{\tau}^{2})\left[\frac{\mathbf{J}_{\mathbf{p}}}{-\mathbf{J}_{\boldsymbol{\tau}}^{\mathbf{T}}\mathbf{J}_{\mathbf{p}}}\right]\left(\mathbf{J}_{\mathbf{p}}^{\mathbf{T}}\mathbf{J}_{\mathbf{p}}\right)^{-1} \qquad \text{Eq. S14}$$

## F. Comparison Between Referenced and Unreferenced Data

Table S1 shows results of model fitting to the isotropic response of 200 mM MeSCN in $H_2O$ for both referenced and unreferenced experimental data. We only collected data up to 10 ps in waiting time, and therefore, we held the vibrational lifetime (known to be ~30 ps) constant during the fit. Parameters between the two data sets agree surprisingly well which shows that model fitting is still reliable for unreferenced data dominated by spectrally correlated shot-to-shot intensity fluctuations. However, the 10× smaller error bars on edge-pixel referenced data clearly show the benefits of removing correlated noise from the data prior to fitting. Figure S2 shows a comparison of FIDs ($\mathrm{T_W} = 0.5$ ps) for both data sets. As seen from the data, the unreferenced noise is comparable in magnitude to the signal.

| # | Parameter | Type | Fitted Value (Referenced Data) | Fitted Value (Unreferenced Data) |
|---|---|---|---|---|
| **1** | $\mathrm{A_{01}}$ | variable | $6.39 \pm 0.01 \times 10^{-5}$ | $8.0 \pm 0.1 \times 10^{-5}$ |
| **2** | $\mathrm{A_{12}}$ | variable | $6.61 \pm 0.01 \times 10^{-5}$ | $5.6 \pm 0.1 \times 10^{-5}$ |
| **3** | $\omega_{01}$ | variable | $2163.438 \pm 0.004$ | $2163.56 \pm 0.03$ |
| **4** | $\delta\omega_1$ | variable | $-0.424 \pm 0.005$ | $-0.57 \pm 0.04$ |
| **5** | $\Delta_{\mathrm{Anh}}$ | variable | $25.846 \pm 0.007$ | $25.84 \pm 0.06$ |
| **6** | $\beta$ | variable | $1.200 \pm 0.002$ | $0.98 \pm 0.01$ |
| **7** | $\mathrm{T_{LT}^{-1}}$ | constant | 0.03 | 0.03 |
| **8** | $\mathrm{T_{Hom}^{-1}}$ | variable | $0.22 \pm 0.004$ | $0.26 \pm 0.04$ |
| **9** | $\tau$ | variable | $1.03 \pm 0.009$ | $0.94 \pm 0.07$ |
| **10** | $\Delta^2$ | variable | $32.1 \pm 0.3$ | $36 \pm 3$ |

Table S1: Results of model fitting to edge-pixel referenced and unreferenced data.

We note that these results differ from those published by Yuan and Fayer[1] ($\tau_1$= 0.4 ps, $\tau_2$ = 1.7 ps, $\Delta_1^2$ = 33.64 cm$^{-2}$, $\Delta_2^2$ = 6.76 cm$^{-2}$, $T_{Hom}^{-1}$ = 0.3297 ps$^{-1}$). The differences in results are reminiscent of the those between our 2020 and 2021 data of MeSCN in DMSO shown in the main text. That is, we did not find convergence with a two-Kubo model even though the CLS in 2020 data suggests two components, and the Kubo time constant obtained by model fitting with a one-Kubo model is ~30% faster than reported by Yuan and Fayer. We propose that measuring the CLS decay as a function of pump power might resolve these inconsistencies.

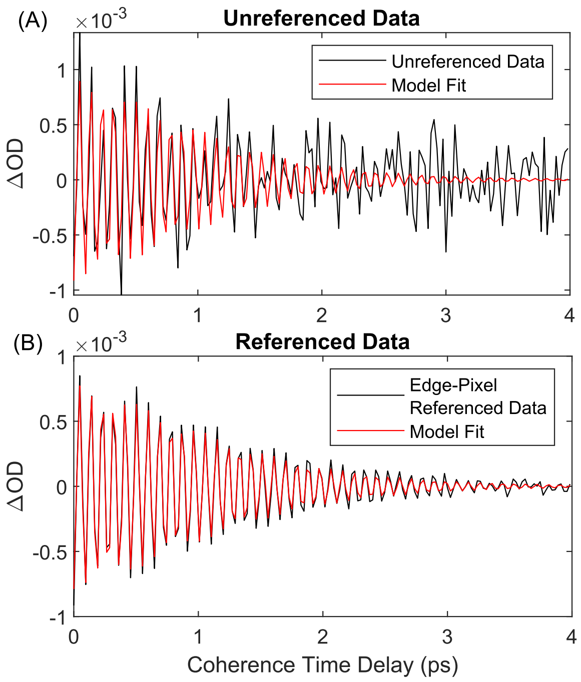


Figure S2. FIDs of unreferenced (A) and edge-pixel referenced (B) data in black. Corresponding model fits are overlayed in red.

## G. Model Fitting with Phasing Errors

We test the effect of phasing errors on model fitting by repeating the simulated Calmodulin experiment, shown in Figure 5 of the main text, with a random phasing error applied to each separate trial (out of 100 trials) sampled from a Gaussian distribution with a standard deviation of 6°, which is roughly the accuracy reported by Backus *et al.*[8] To better distinguish the effect of phasing errors from regular noise, we also increase the SNR to 100:1. Fitting trajectories in Video S10 show that, without a phase correction fitting parameter, model fitting yields reasonable accuracy with the exception of $\omega_{01}$ (the 0-1 center frequency), which varies ±1 cm$^{-1}$ to adjust for the phasing errors. It is important to enable a ±2 cm$^{-1}$ range for the pump axis calibration error $\delta\omega_1$ here to avoid boundary stalling since $\delta\omega_1$ works to counteract the ~1 cm$^{-1}$ deviations $\omega_{01}$ along the pump axis Unfortunately, we couldn't find room to fit $\delta\omega_1$ on the fitting trajectories plots, so this insight came from manually looking at the numerical results. The MATLAB code for reproducing this data is freely available with instructions below. Updates of dephasing parameters are shown for every trial in Video S11. Unfortunately, these results show skewed distributions of fitting parameters in panel (C) and notable underestimates of 95% confidence intervals in panel (D), which implies nonlinear propagation of error. In other words, confidence intervals are unreliable when model fitting to data with phasing errors while foregoing a fitting parameter to account for phasing errors. On the other hand, fitting parameter values are, on average, surprisingly accurate as shown in panel (E).

We now introduce a fitting parameter $\phi$ to account for phasing errors in a similar manner to Garrett-Roe and coworkers.[9-11] Fitting trajectories in Video S12 show that model fitting yields accurate fits of all fitting parameters, including $\omega_{01}$ now. Video S13 shows updates of dephasing parameters for every trial. The results show symmetric distributions of fitting parameters in panel

(C) and reliable estimates of 95% confidence intervals in panel (D), which together imply that error propagates linearly when including a phase correction fitting parameter. Further results in panel (E) show accurate fits of dephasing parameters over the course of 100 fitting trials.

It should be noted that this model assumes phase stability over the course of the entire experiment, where the phase error is the same for every waiting time spectrum, and equally symmetric for the rephasing and nonrephasing spectrum (i.e. $e^{\pm i\phi}$ in Eq. S2). In practice, this level of phase stability requires considerable effort to maintain with four-wave-mixing apparatus. Where these assumptions do not apply, we cannot guarantee the accuracy or reliability of model fitting. While there are many factors that can cause phase instability, the presence of spectrally correlated shot-to-shot noise (a.k.a. local-oscillator noise) further complicates the post-processing procedure for correcting phase errors, particularly with the projection slice theorem. Therefore, we do not recommend model fitting for phase distorted apparatuses without prior removal of shot-to-shot noise (e.g. using a calibrated referencing scheme[3, 12, 13]).

## H. Instructions for Reproducing Results and Figures

The standalone desktop app (available for Windows and Mac users), MATLAB source code, and experimental data are freely available at https://github.com/kevin-robben/model-fitting. The following instructions are provided for reproducing results and figures. Note that MATLAB R2020 or newer is required. Scripts concerning simulated data have the random number generators initialized with seed = 1 (i.e. `rng(1)`), which enables exact reproducibility of results. Likewise, all experimental data is included.

Figure 1: Run the following scripts in order:
1. Sim MeSCN H2O\fit_100_trials.m
2. Sim MeSCN H2O\makeVideos.m (if videos are desired)

Figure 2: Run the script: "CLS Propagation of Error\VIF_comparison.m"

Figure 3: This figure is pieced together from a few different scripts.
Plots on the left are obtained by running the script: "Sim Under param\fit_under_param.m"
Plots in the middle are also obtained when generating Figure 1.
Plots on the right are obtained by running the script: "Sim Over param\fit_over_param.m"

Figure 4: Results similar to this figure can be obtained by running the scripts to reproduce the right column of Figure 3, and then copying the plots from any of the figures saved in the folder: "Sim Over param\Output Data\Fitting Update Figures\"

Figure 5: Run the following scripts in order:
1. Sim CNC in Calmodulin - Low SNR\fit_100_trials_low_SNR.m
2. Sim CNC in Calmodulin - Low SNR\makeVideos.m

Figures 6 - 12: Results are reproduced by running the following scripts in order:
1. Exp MeSCN DMSO - 2020 Data\fit_1kubo_MeSCN_DMSO_2020.m
2. Exp MeSCN DMSO - 2020 Data\CLS_analysis_2020.m
3. Exp MeSCN DMSO - 2020 Data\make2020TwSeriesComparisonVideo.m

4. Exp MeSCN DMSO - 2020 Data\fit_2kubo_MeSCN_DMSO_2020.m
5. Exp MeSCN DMSO - 2021 Data\fit_MeSCN_DMSO_2021.m
6. Exp MeSCN DMSO - 2021 Data\fitting_analysis_2021.m
7. Exp MeSCN DMSO - 2021 Data\make2021TwSeriesComparisonVideo.m

Figure 6 is located at:
Exp MeSCN DMSO - 2021 Data\Output Data\CLS Log Axis.fig
Plots in Figure 7 are taken from:
Exp MeSCN DMSO - 2020 Data\Output Data 1-Kubo\C and SIGN.fig
Exp MeSCN DMSO - 2020 Data\Output Data 2-Kubo\C and SIGN.fig
Plots in Figure 8 are taken from:
Exp MeSCN DMSO - 2021 Data\Output Data\Linear Absorbance.fig
Plots in Figure 9 are taken from:
Exp MeSCN DMSO - 2020 Data\Output Data 1-Kubo\comparison plot at 400 fs.fig
Exp MeSCN DMSO - 2020 Data\Output Data 1-Kubo\comparison plot at 50 ps.fig
Figure 10 is located at:
Exp MeSCN DMSO - 2021 Data\Output Data\Sampling Masks.fig
Figure 11 is located at:
Exp MeSCN DMSO - 2021 Data\Output Data\C and SIGN.fig
Figure 12 is located at:
Exp MeSCN DMSO - 2021 Data\Output Data\Params.fig

Eq. S10 is checked using the following steps:

3. Open the model fitting GUI located at "Model Fitting GUI\Model_Fitting_GUI.mlapp"
4. Click the "Model and Fitting" tab, then the "Model" subtab, and then "Load From File".
5. From the folder "Model Fitting GUI\Input Data\", select and open one of the available models (e.g. "p 1-kubo.csv").
6. Click the "Simulation Axes" tab, then add several Tw points by clicking the "Add Tw Point" button several times and edit the "Tw (ps)" field in the table to assign specific Tw times. You may edit other fields too, or just stick with the defaults and move on.
7. Navigate up one folder to "Manuscript Examples\Exp MeSCN H2O\" and select "p init guess.csv".
8. Go back to the "Model and Fitting" tab, and then the "Simulation" subtab. Click the "Copy From Model" button, and then the "Simulate Data" button.
9. Now click the "Start Fitting" button. After the fitting is complete, it will default to the "Best Fit" subtab.
10. Move over to the "Compare (Simulation Only)" subtab. This subtab actually updates the moment you press "Simulate Data", so you could really look at it at any time, but the "Actual Fit Value" column won't update until after fitting. The column "Simulated (True) Value" corresponds to $\mathbf{p}^*$, the column "Predicted Fit Value" corresponds to $\mathbf{p}^* + \mathbf{dp}$ (where $\mathbf{dp}$ is from Eq. S10), and the column "Actual Fit Value" corresponds to the actual fitting parameters just determined. Compare the predicted fit values to the actual fit values. You should find that they agree quite well for reasonable circumstances of noise and lineshape models.

Eq. S13 is verified by running the script:

Manuscript Experiments\CLS Propagation of Error\TestSIEq13.m

The data from Table S1 is reproduced as follows:

1. Open the model fitting GUI located at "Model Fitting GUI\Model_Fitting_GUI.mlapp"
2. Go to File → Load → Data → MATLAB-Ready Format
3. Navigate to "Manuscript Examples\Exp MeSCN H2O\Edge-Pixel Referenced\" and select "Exp MeSCN in H2O - Edge-Pixel Referenced (MATLAB-Ready).mat"
4. Click the "Model and Fitting" tab, then the "Model" subtab, and then "Load From File".
5. Navigate up one folder to "Manuscript Examples\Exp MeSCN H2O\" and select "p init guess.csv".
6. Click "Start Fitting". Because there's 167 points along the pump axis, this may take 10 minutes or longer to complete (therefore we suggest larger step sizes with fewer points along the pump axis).
7. After fitting is complete, the program should automatically switch to the "Best Fit" subtab. Click "Save to File" to save results to a csv file to review.
8. Steps 1-7 produce edge-pixel referencing data. For unreferenced data, repeat steps 3-7 while navigating to the "Unreferenced" folder.

Phasing error simulations and fitting results are reproduced by running the following scripts:

1. Sim Phasing Error without Variable phi\fit_phase_error.m
2. Sim Phasing Error without Variable phi\MakeVideos.m
3. Sim Phasing Error with Variable phi\fit_phase_error.m
4. Sim Phasing Error with Variable phi\MakeVideos.m

## I. References


1. Yuan, R.; Fayer, M. D., Dynamics of water molecules and ions in concentrated lithium chloride solutions probed with ultrafast 2D IR spectroscopy. *The Journal of Physical Chemistry B* **2019,** *123* (35), 7628-7639.
2. Schmidt-Engler, J. M.; Zangl, R.; Guldan, P.; Morgner, N.; Bredenbeck, J., Exploring the 2D-IR repertoire of the–SCN label to study site-resolved dynamics and solvation in the calcium sensor protein calmodulin. *Physical Chemistry Chemical Physics* **2020,** *22* (10), 5463-5475.
3. Robben, K. C.; Cheatum, C. M., Edge-pixel referencing suppresses correlated baseline noise in heterodyned spectroscopies. *The Journal of Chemical Physics* **2020,** *152* (9), 094201.
4. Marple, L., Computing the discrete-time" analytic" signal via FFT. *IEEE Transactions on signal processing* **1999,** *47* (9), 2600-2603.
5. Nocedal, J.; Wright, S., *Numerical optimization*. Springer Science & Business Media: 2006.
6. Rector, K.; Kwok, A.; Ferrante, C.; Tokmakoff, A.; Rella, C.; Fayer, M., Vibrational anharmonicity and multilevel vibrational dephasing from vibrational echo beats. *The Journal of chemical physics* **1997,** *106* (24), 10027-10036.
7. Hamm, P.; Zanni, M. T., *Concepts and methods of 2d infrared spectroscopy*. Cambridge University Pres: Cambridge ; New York, 2011; p ix, 286 p.
8. Backus, E. H.; Garrett-Roe, S.; Hamm, P., Phasing problem of heterodyne-detected two-dimensional infrared spectroscopy. *Optics letters* **2008,** *33* (22), 2665-2667.

9. Johnson, C. A.; Parker, A. W.; Donaldson, P. M.; Garrett-Roe, S., An ultrafast vibrational study of dynamical heterogeneity in the protic ionic liquid ethyl-ammonium nitrate. I. Room temperature dynamics. *The Journal of Chemical Physics* **2021,** *154* (13), 134502.
10. Brinzer, T.; Berquist, E. J.; Ren, Z.; Dutta, S.; Johnson, C. A.; Krisher, C. S.; Lambrecht, D. S.; Garrett-Roe, S., Ultrafast vibrational spectroscopy (2D-IR) of CO2 in ionic liquids: Carbon capture from carbon dioxide's point of view. *The Journal of chemical physics* **2015,** *142* (21), 212425.
11. Ren, Z.; Brinzer, T.; Dutta, S.; Garrett-Roe, S., Thiocyanate as a Local Probe of Ultrafast Structure and Dynamics in Imidazolium-Based Ionic Liquids: Water-Induced Heterogeneity and Cation-Induced Ion Pairing. *The Journal of Physical Chemistry B* **2015,** *119* (13), 4699-4712.
12. Feng, Y.; Vinogradov, I.; Ge, N. H., General noise suppression scheme with reference detection in heterodyne nonlinear spectroscopy. *Optics Express* **2017,** *25* (21), 26262-26279.
13. Feng, Y.; Vinogradov, I.; Ge, N. H., Optimized noise reduction scheme for heterodyne spectroscopy using array detectors. *Optics Express* **2019,** *27* (15), 20323-20346.
